# Diverse somatic genomic alterations in single neurons in chronic traumatic encephalopathy

**DOI:** 10.1126/science.adu1351

**Published:** 2025-10-30

**Authors:** Guanlan Dong, Chanthia C. Ma, Shulin Mao, Katherine Sun-Mi Brown, Samuel M. Naik, Gannon A. McDonough, Samadhi P. Wijethunga, Junho Kim, Samantha L. Kirkham, Diane D. Shao, Jonathan D. Cherry, Madeline Uretsky, Elizabeth Spurlock, Ann C. McKee, August Yue Huang, Michael B. Miller, Eunjung Alice Lee, Christopher A. Walsh

**Affiliations:** 1 Division of Genetics and Genomics, Manton Center for Orphan Disease Research, Boston Children’s Hospital; Boston, MA 02115, USA.; 2 Department of Pediatrics, Harvard Medical School; Boston, MA 02115, USA.; 3 Bioinformatics and Integrative Genomics Program, Harvard Medical School; Boston, MA 02115, USA.; 4 Harvard-MIT MD-PhD Program, Harvard Medical School; Boston, MA 02115, USA.; 5 Program in Biological and Biomedical Sciences, Harvard Medical School; Boston, MA 02115, USA.; 6 Division of Neuropathology, Department of Pathology, Brigham and Women’s Hospital, Harvard Medical School; Boston, MA 02115, USA.; 7 Department of Biological Sciences, Sungkyunkwan University; Suwon 16419, South Korea.; 8 Department of Neurology, Boston Children’s Hospital; Boston, MA 02115, USA.; 9 Veterans Affairs (VA) Boston Healthcare System, US Department of Veteran Affairs; Boston, MA 02132, USA.; 10 Alzheimer’s Disease Research Center and Chronic Traumatic Encephalopathy Center, Chobanian and Avedisian School of Medicine, Boston University; Boston, MA 02118, USA.; 11 Department of Pathology and Laboratory Medicine, Chobanian and Avedisian School of Medicine, Boston University; Boston, MA 02118, USA.; 12 Department of Neurology, Chobanian and Avedisian School of Medicine, Boston University; Boston, MA 02118, USA.; 13 Broad Institute of MIT and Harvard; Cambridge, MA 02142, USA.; 14 Howard Hughes Medical Institute; Boston, MA 02115, USA.

## Abstract

Chronic traumatic encephalopathy (CTE) is a neurodegenerative disease linked to exposure to repetitive head impacts (RHI), yet little is known about its pathogenesis. Applying two single-cell whole-genome sequencing methods to hundreds of neurons from prefrontal cortex of 15 individuals with CTE, and 4 with RHI without CTE, we revealed increased somatic single-nucleotide variants in CTE, exhibiting a pattern previously reported in Alzheimer’s disease (AD). Furthermore, we discovered high burdens of somatic small insertions and deletions in a subset of CTE individuals, resembling a known pattern, ID4, also found in AD. Our results suggest that neurons in CTE experience stereotyped mutational processes shared with AD; the absence of similar changes in RHI neurons without CTE suggests that CTE involves mechanisms beyond RHI alone.

Chronic traumatic encephalopathy (CTE) develops years after exposure to repetitive head impacts (RHI) and is most often found in athletes playing contact sports (1–5). CTE is diagnosed by postmortem neuropathological examination, based on the presence of a pathognomonic lesion consisting of perivascular hyperphosphorylated tau neurofibrillary tangles in neurons at the depths of the cortical sulci (1, 2). Tau deposition is common to CTE and Alzheimer’s disease (AD) (4, 6); however, CTE is characterized by distinct neuropathological features, tau molecular structural conformation (7, 8), and clinical symptoms (5, 9). The precise mechanisms by which RHI induces tau neurodegeneration are poorly understood. Somatic mutations are well-known drivers of cellular proliferation in neoplasia (10–12), but also accumulate in non-neoplastic cells and even non-dividing neurons with age (13–17). Multiple studies using single-cell whole-genome sequencing (scWGS) (17, 18) and single-molecule duplex sequencing (16, 19, 20) converge on accumulation rates of 16–17 somatic single-nucleotide variants (sSNVs) per year and 2–3 somatic small insertions and deletions (sIndels) per year in neurons from neurotypical controls. Furthermore, neurons from individuals with AD and other neurodegenerative disorders show higher sSNV burden with distinct mutational signatures (18, 21). Here we applied scWGS to amplified DNA from single neuronal nuclei isolated from CTE, RHI without CTE, AD, and neurotypical control individuals (Fig. 1A), using both strand-agnostic and strand-specific amplification methods to assess somatic mutations in CTE.

## sSNVs in CTE neurons

**Fig. 1.**
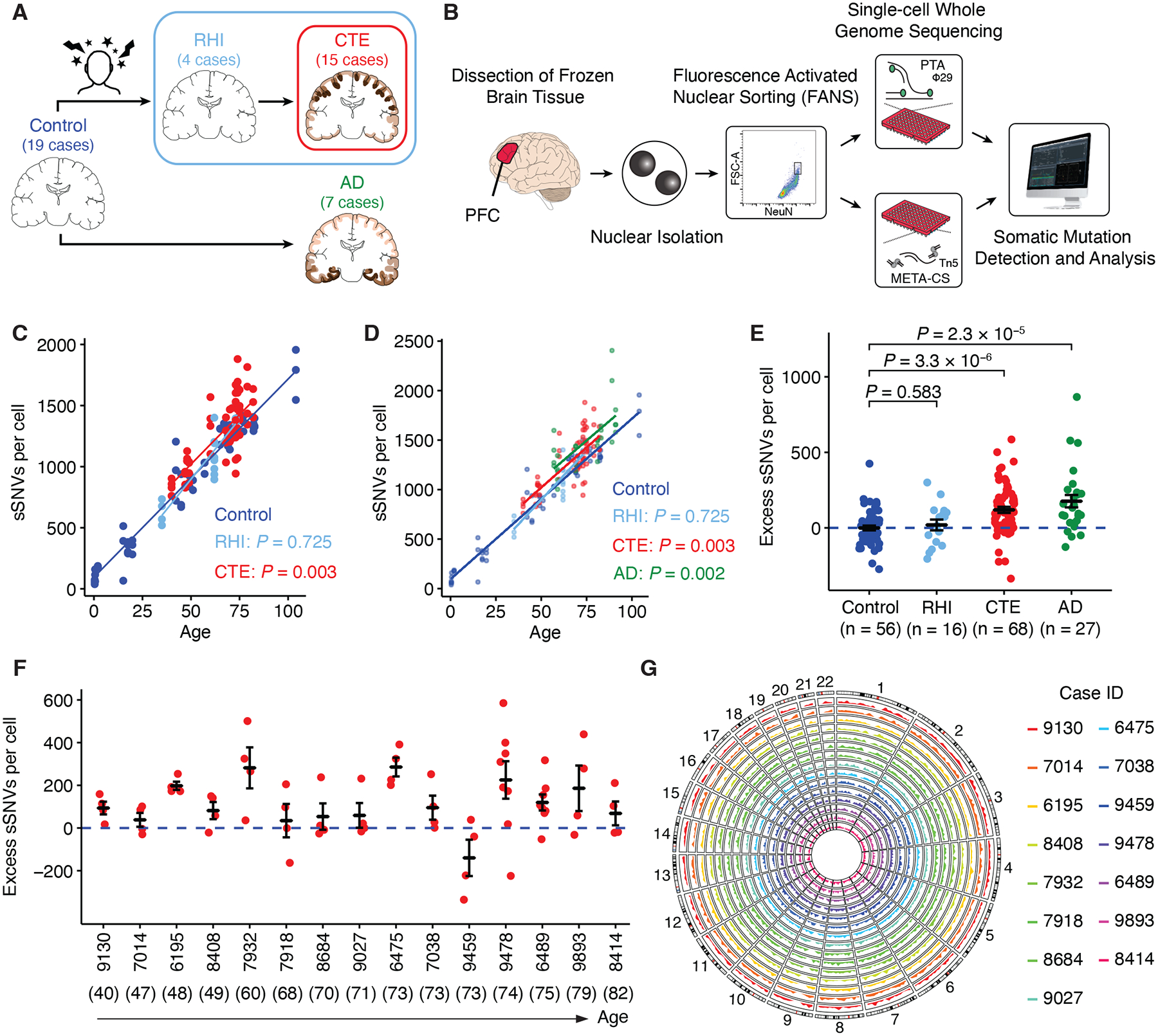
Study design and sSNV burden in CTE neurons. (**A**) Cohort design: neurotypical control, repetitive head impacts (RHI), chronic traumatic encephalopathy (CTE), and Alzheimer’s disease (AD). Illustrations of CTE and AD show characteristic tau pathology patterns (in brown). The number of cases included in this study are shown for each clinical condition. (**B**) Single-cell whole-genome sequencing (WGS) experimental approach. Nuclei are isolated from postmortem human brain tissue and subjected to fluorescence-activated nuclear sorting for the neuronal nuclear marker NeuN. Nuclei are sorted, lysed, and subjected to primary template amplification (PTA) and multiplexed end-tagging amplification of complementary strands (META-CS). Amplified genomic DNA is then assayed using WGS to identify somatic mutations. PTA data is used to determine somatic mutation burden, while META-CS data is used to identify strand-related signatures. (**C**) sSNV burden in CTE (red), RHI (light blue), and neurotypical control brains (dark blue) with a significant increase in CTE but not in RHI when compared to controls. sSNV burden (from each neuron as a point) estimated by SCAN2 is fitted against age by clinical conditions using LME models (neurotypical control: dark blue; RHI: light blue, *P* = 0.725; CTE: red, *P* = 0.003). P-values compare each clinical condition against controls. (**D**) Similar to (C) with added neurons from AD (green) brains (AD: green, *P* = 0.002 using the LME model). P-values compare each clinical condition against controls. (**E**) Excess sSNV burden in RHI, CTE, and AD compared to neurotypical control after adjusting for age. Data are mean ± standard error. The dashed blue line shows sSNVs attributable to age (zero excess). P-values are from two-tailed Wilcoxon tests. (**F**) Excess sSNVs in each CTE case ordered by increasing age (indicated in parentheses). The dashed blue line shows sSNVs attributable to age (zero excess). (**G**) Circos plot showing the PTA sSNV density distribution of CTE cases across the whole genome. Each CTE case is depicted by color in a circular track.

We acquired frozen brain tissue from dorsolateral prefrontal cortex (PFC) from individuals with a history of RHI, with and without CTE, as well as neurotypical controls. We isolated nuclei of single neurons by staining for the nuclear stain DAPI, the pan-neuronal marker NeuN, and sorted neurons using fluorescence-activated nuclei sorting (FANS), gating specifically for the largest NeuN-positive nuclei (Fig. 1B, fig. S1). This gating method isolates pyramidal excitatory neurons with a purity of greater than 99% (18). We sorted single nuclei into individual wells of 96-well plates, then performed whole-genome amplification using primary template amplification (PTA) (22) or multiplexed end-tagging amplification of complementary strands (META-CS) (19). Amplified DNA underwent multiple screening and quality control steps, ensuring that only well-amplified genomes were used for sequencing. We sequenced PTA-amplified genomes of 68 neurons from 15 cases of CTE (exposed to RHI with neuropathologically verified CTE), 16 neurons from 4 cases of RHI (exposed to RHI without CTE), and compared these with 56 neurons from 19 neurotypical controls (23) and 27 neurons from 7 cases of AD (18) (table S1–3).

We identified both sSNVs and sIndels in each single PTA neuron using SCAN2 (17) and found significantly increased sSNVs in CTE, averaging 114 more sSNVs per cell compared to controls (*P* = 0.003, linear mixed-effects (LME) model; Fig. 1C). The increased sSNV burdens are comparable to those in AD (Fig. 1D). In contrast, sSNV numbers in RHI cases were indistinguishable from controls (*P* = 0.725, LME model; Fig. 1C), suggesting that increased sSNVs in CTE arise not from RHI exposure alone, but from additional factors associated with the development of CTE. All CTE and RHI brain samples were collected and processed at the UNITE Brain Bank to control for potential batch effects, though the small sample size of RHI may limit statistical power. We performed non-parametric tests on somatic burdens after adjusting for age, where both CTE and AD neurons showed a significant excess of sSNVs compared to controls (CTE: *P* = 3.3 × 10^−6^, AD: *P* = 2.3 × 10^−5^, two-tailed Wilcoxon test; Fig. 1E and fig. S2A–C). The increased sSNVs in CTE remained significant after controlling for additional quality metrics (see Methods, fig. S3). Furthermore, although neurons in each case showed some variation, we observed greater mean sSNVs in nearly all CTE cases than the burden attributable to normal aging (Fig. 1F).

Somatic mutations were broadly distributed across each neuron’s genome (Fig. 1G). Interestingly, we found no association between sSNV burden and potential risk factors for CTE pathology, including cortex topology (1) (fig. S4) and years of playing American football (6, 24–26), which implies that the duration of RHI exposure does not directly lead to increased sSNVs, in line with the similar sSNV burden between RHI and controls.

To investigate the strand-specificity of excess sSNVs in CTE, we used a modified duplex META-CS method (19) (see Methods) to profile 115 neurons from 12 CTE cases and 146 neurons from 16 neurotypical controls with paired PTA data (table S1). We distinguished variants representing double-stranded mutations and single-stranded DNA lesions, respectively (fig. S5A). According to META-CS-derived strand-specific signatures, PTA-profiled sSNVs showed on average 85% double-stranded signature contribution (fig. S5B), indicating that PTA-identified sSNVs are predominantly double-stranded, albeit not exclusively. This aligns with the highly similar rate of age-related sSNV increase when profiled by either PTA (17) or strand-specific methods (16, 19).

## Mutational signatures of sSNVs in CTE neurons

**Fig. 2.**
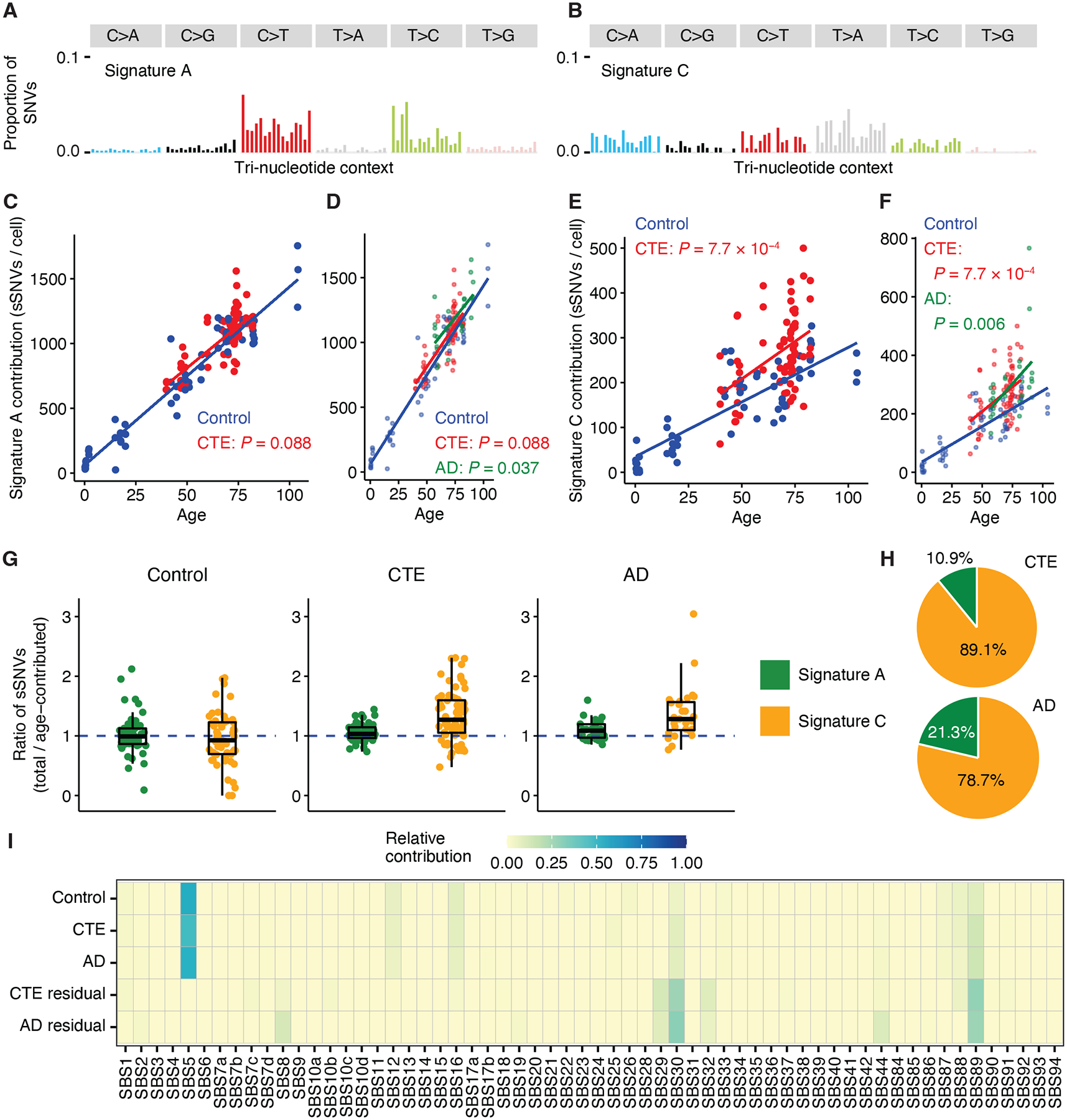
sSNV mutational signatures in CTE neurons. (**A, B**) Mutation spectra of Signatures A and C. **(C-F)** sSNV burden separated by contributions from Signature A (C, D) and Signature C (E, F) in CTE (red), AD (green), and neurotypical control (dark blue) neurons. These signatures decompose sSNV burden into age- and disease-specific effects. Their contributions are fitted against age by clinical conditions using LME models (C, Signature A, CTE: *P* = 0.088; D, Signature A, CTE: *P* = 0.088, AD: *P* = 0.037; E, Signature C, CTE: *P* = 7.7 × 10^−4^; F, Signature C, CTE: *P* = 7.7 × 10^−4^, AD: *P* = 0.006). P-values compare each clinical condition against controls. (**G**) Ratios of each signature’s total contribution to age-related contribution in neurotypical control, CTE, and AD neurons. Age-contributed sSNVs of each signature are obtained from the LME model in neurotypical controls. A ratio > 1 indicates a higher contribution from the signature compared to age-matched controls. Bars in each box plot from top to bottom show the first, second (median), and third quartile; whiskers extend 1.5 interquartile range (IQR). (**H**) Relative contribution of Signatures A and C in CTE and AD after adjusting for age. Based on the ratios shown in (G), the relative contribution of each signature is calculated by removing the median ratio of neurotypical controls (representing age effect) from the median ratio of each disease. (**I**) Mutational spectra of neurotypical control, CTE, and AD neurons are fitted to the COSMIC SBS database of cancer mutational signatures. Residual mutational patterns for CTE and AD are obtained by subtracting mutation profiles of age-matched controls from those of CTE and AD to show disease-specific contributions.

To investigate potential sources of sSNVs in CTE neurons, we performed mutational signature analysis on the PTA scWGS data from CTE and neurotypical control neurons. First, we decomposed the sSNVs into Signatures A and C, previously identified in single neurons (21) (Fig. 2A, B). Signature A closely resembles SBS5 from the COSMIC database (v.3.2) (27), a clock-like signature associated with aging. Signature C resembles SBS8, a signature associated with deficient transcription-coupled nucleotide excision repair (TC-NER) (21, 28), along with other SBS signatures, which have also been linked with oxidative damage in AD neurons (18). The contribution of Signature A increased with age without showing differences between CTE and control neurons (Fig. 2C, D). In contrast, we observed a significantly larger contribution of Signature C in CTE neurons, with an average excess of 64 sSNVs per cell (*P* = 7.7 × 10^−4^, LME model; Fig. 2E, F). We also compared the signature contributions to 27 previously reported AD neurons (18), in which AD showed a greater contribution of both Signature A and Signature C compared to age-matched controls (*P* = 0.037 and 0.006, LME model; Fig. 2D, F).

To better understand the age- and disease-associated contributions of the two signatures, we calculated the ratio of each signature’s total sSNVs to age-contributed sSNVs and found an elevated contribution of Signature C (ratio > 1) in CTE and AD (Fig. 2G), suggesting shared mutational mechanisms in both neurodegenerative tauopathies. When we compared the relative contribution of the two signatures after adjusting for age, we found that Signature C accounted for a greater proportion of the increase in CTE than in AD (Fig. 2H). META-CS data identified a mean contribution of Signature C ranging from 22–24% in double-stranded SNVs (dsSNVs) and around 67% in single-stranded SNVs (ssSNVs) without differences between CTE and age-matched controls (fig. S6). This finding, combined with the predominant contribution of dsSNVs (~85%) in PTA neurons, suggests that a substantial amount of Signature C is double-stranded. Although the precise mechanism of Signature C and SBS8-like variants has not been definitively determined, the C>A mutations in this pattern are associated with alterations in reactive oxygen species (ROS) in other contexts (29) and represent one possible contributor to disease pathogenesis.

We also conducted *de novo* signature analysis using non-negative matrix factorization (NMF) (30) (see Methods). We identified two *de novo* signatures, N1 and N2, which shared many similarities with Signatures C and A, respectively, but also had substantial overlaps with each other (such as SBS5 and SBS16) (fig. S7) which may limit their interpretability as they may not represent distinct biological processes. To investigate other potential contributing factors to the excess sSNVs in CTE, we aggregated mutations based on their disease status (neurotypical control, CTE, and AD) and fitted them to COSMIC SBS signatures (Fig. 2I). The aging effect represented by SBS5 dominated across all three groups. However, after subtracting the pattern of age-matched controls from CTE and AD, we obtained residual patterns that were disease-specific (Fig. 2I and fig. S7D), suggesting elevated contributions of SBS29 and SBS32 in CTE that are almost absent in controls. SBS29 is a signature associated with tobacco chewing, yet distinct from tobacco smoking-related SBS4 and SBS92 (27). A high prevalence of smokeless tobacco usage has been reported in athletes (31, 32); smokeless tobacco usage in American football players is unknown. SBS32 is associated with azathioprine treatment and was recently reported to be strongly associated with age in oligodendrocytes with an absence in aging neurons (23). Other COSMIC signatures, including SBS30 and SBS89, were present in controls but increased in CTE; SBS30 contributed to Signature C (fig. S7C) and was associated with deficient base excision repair which may also play a role in the excessive DNA damage, whereas the mechanism underlying SBS89 is unclear.

## sIndels in CTE neurons

**Fig. 3.**
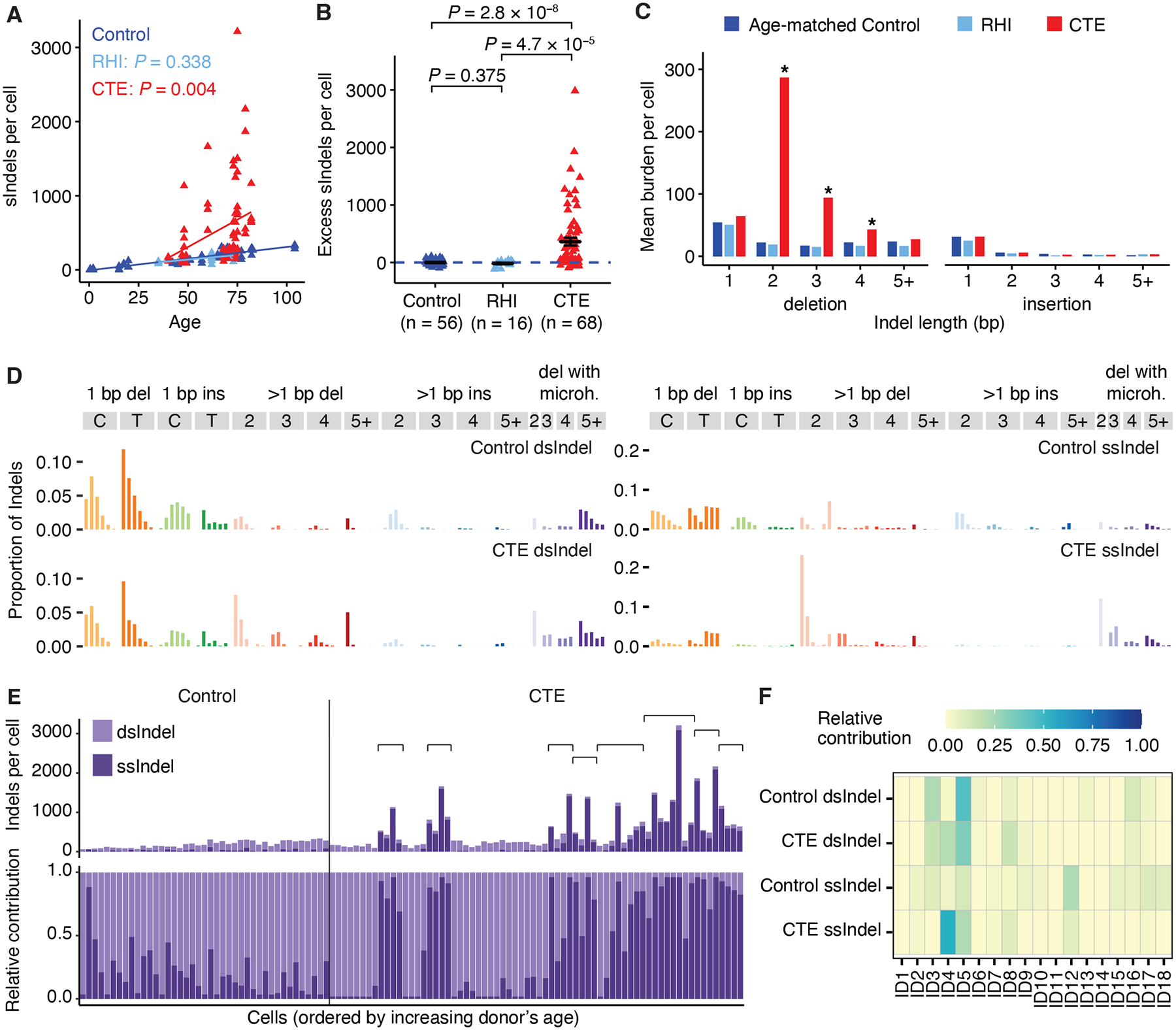
sIndel burden and mutational signatures in CTE neurons. sIndel burdens were identified from PTA scWGS data, with strand-related analysis inferred from mutational signatures extracted from META-CS scWGS data. (**A**) sIndel burden in CTE (red), RHI (light blue), and neurotypical control brains (dark blue) with a significant increase in CTE but not in RHI when compared to controls. sIndel burden (from each neuron as a triangle) estimated by SCAN2 is fitted against age by clinical conditions using LME models (neurotypical control: dark blue; RHI: light blue, *P* = 0.338; CTE: red, *P* = 0.004). P-values compare each clinical condition against controls. (**B**) Excess sIndel burden in RHI and CTE compared to neurotypical control after adjusting for age. Data are mean ± standard error. The dashed blue line shows sIndels attributable to age (zero excess). P-values are from two-tailed Wilcoxon tests. (**C**) Comparison of all types of sIndels across age-matched controls, RHI, and CTE. Data are mean burden per cell. Asterisk denotes significant changes in certain types of sIndels when compared to age-matched controls (*P* < 0.05, two-tailed Wilcoxon test). (**D**) Double-stranded (ds) and single-stranded (ss) Indel signatures in neurotypical controls and CTE extracted from META-CS data. (**E**) Absolute (*top*) and relative (*bottom*) contribution of dsIndel and ssIndel signatures in PTA-profiled neurotypical control and CTE neurons. The pair of dsIndel and ssIndel signatures used for decomposition is determined by the clinical condition of PTA neurons. CTE cases with a pronounced ssIndel contribution are indicated by brackets. Cells with < 15 Indels are not shown. (**F**) Decomposition of dsIndel and ssIndel signatures to COSMIC ID database.

Compared to sSNVs, sIndels showed a more pronounced increase in CTE than in controls (*P* = 0.004, LME model; *P* = 2.8 × 10^−8^, two-tailed Wilcoxon test; Fig. 3A, B and fig. S2D–F). RHI neurons showed no differences in sIndel burden versus controls, further supporting the hypothesis that increased somatic burden is specific to CTE pathology. CTE neurons accumulated a mean of 312 excess sIndels per cell versus controls, approximately three times the CTE-specific sSNV increase and equivalent to sIndels accumulated over > 100 years (given the normal neuronal rate of 2–3 sIndels per year per genome). These CTE excess sIndels are predominantly 2 to 4 bp deletions (Fig. 3C). Re-analysis of PTA AD neurons from our previous study (18) revealed a similar phenomenon in AD (fig. S8).

We developed a new computational pipeline (see Methods) specifically for identifying sIndels in single-cell META-CS data and extracted mutational signatures that represented double-stranded and single-stranded Indels (dsIndels and ssIndels) in CTE and neurotypical controls (Fig. 3D). We confirmed the robustness of these signatures with different calling thresholds (fig. S9A–D). After fitting the dsIndel and ssIndel signatures to PTA-profiled sIndels (see Methods), we found that the excess sIndels in some CTE individuals are primarily single-stranded, whereas other CTE individuals and controls have more modest numbers of sIndels that are primarily double-stranded (Fig. 3E).

To explore potential biological processes giving rise to dsIndels and ssIndels in CTE, we decomposed META-CS-derived signatures into COSMIC Indel (ID) signatures (v.3.2) (Fig. 3F). Some signatures were present in both CTE and neurotypical controls. ID5, a clock-like signature associated with aging, contributes to dsIndels and at a lesser degree to ssIndels of both groups. ID12, a signature with unknown etiology where Indels occur primarily in repetitive regions, showed a strong contribution to ssIndels of both groups, implying that repetitive regions may be prone to single-stranded lesions or sequencing errors. Since the current technology may not distinguish sequencing errors or amplification artifacts from single-stranded lesions, we focused on differences observed between control and CTE which are more likely to reflect biological processes. ID4, mainly characterized by 2 to 4 bp deletions, has a predominant and robust (fig. S9E–H) presence in CTE and is minimal in controls, consistent with the bias towards 2 to 4 bp deletions observed in CTE PTA neurons (Fig. 3C). Although the signal is more pronounced in the ssIndel signature, the dsIndel signature also showed a substantial ID4 contribution, indicating a mutagenic process where a subset of ID4-associated DNA lesions become double-stranded mutations.

## Somatic ID4-like deletions in certain CTE individuals

**Fig. 4.**
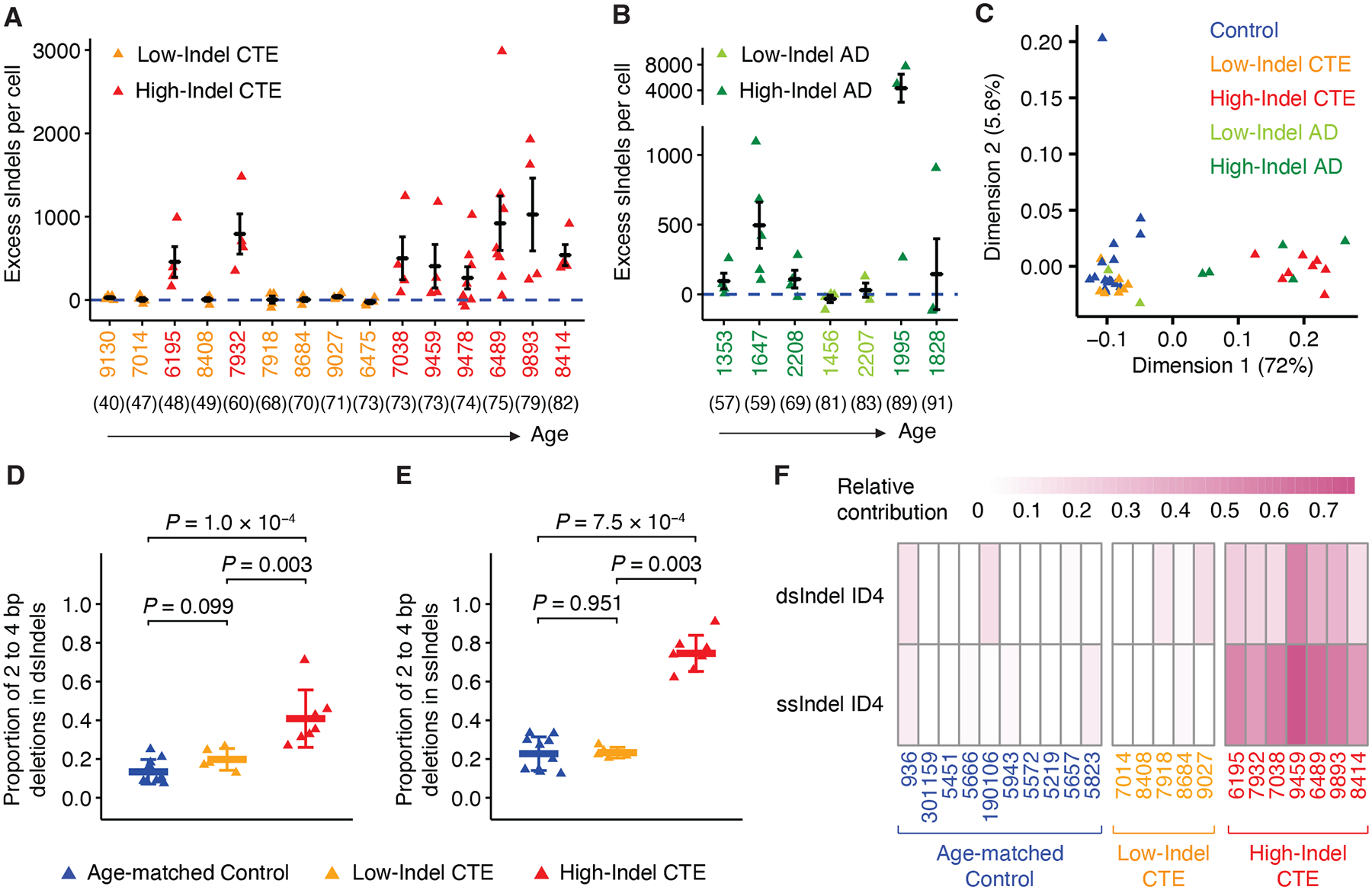
Somatic ID4-like deletions in certain CTE individuals. (**A**, **B**) Excess sIndels in each CTE (A) and AD (B) case ordered by increasing age (parentheses). CTE and AD cases are separated into either High-Indel group (High-Indel CTE: red, High-Indel AD: dark green) or Low-Indel group (Low-Indel CTE: yellow, Low-Indel AD: light green) based on whether they have an excess of sIndels (> 50). Data are mean ± standard error. The dashed blue line shows sIndels attributable to age (zero excess). (**C**) Principal component analysis (PCA) clustering of sIndels from each case across neurotypical controls, CTE, and AD. sIndels from each case were aggregated and stratified into 83 contexts defined by COSMIC. The first two dimensions are used for visualization with the percentage of variance explained shown for each dimension. Cases with < 15 sIndels are not shown. (**D**, **E**) Comparison of proportions of 2 to 4 bp deletions in dsIndels (D) and ssIndels (E) across age-matched controls, Low-Indel CTE, and High-Indel CTE. Each triangle represents an individual from META-CS data. High-Indel CTE vs. Age-matched controls: *P* = 1.0 × 10^−4^ and *P* = 7.5 × 10^−4^, two-tailed Wilcoxon test. Data are mean ± standard deviation. (**F**) Relative contribution of ID4 to dsIndels and ssIndels of each case from META-CS data shown as a heatmap. Case IDs are colored by their group assignment (age-matched control: dark blue, Low-Indel CTE: yellow, High-Indel CTE: red).

The excess sIndels in CTE were driven by a subset of individuals (Fig. 4A), designated High-Indel CTE, whereas other CTE individuals exhibited much lower excess sIndels compared to age-matched controls, designated Low-Indel CTE. This distinction in excess sIndels between High-Indel and Low-Indel CTE groups remained after controlling for quality metrics (see Methods; fig. S10). Although individuals in the High-Indel group were generally older (> 70 years old), two younger individuals suggest that other factors beyond age may contribute. We observed modest correlation between excess sIndels and excess sSNVs in High-Indel CTE at the cellular level, but limited power precludes a clear conclusion (fig. S11). Furthermore, we found a significant association between sIndels in CTE and the duration of symptoms (the time between symptoms onset and death; *P* = 0.022, LME model), which was stronger than the association with age at death (*P* = 0.059, LME model), suggesting that the duration of disease might contribute to excess sIndels. The *APOE* ε4 allele has been identified as a prominent risk factor for AD (33) and is associated with the severity of CTE tau pathology (34), however, we found no association of *APOE* ε4 with sIndels in CTE after controlling for age (see Methods). Years of playing football, a proxy for cumulative RHI exposure, also did not show association, consistent with our findings in sSNV. Re-analysis of PTA AD neurons (18) revealed similarly high rates of sIndels in certain AD individuals (designated High-Indel AD versus Low-Indel AD, Fig. 4B), indicating that excessive sIndels may contribute to multiple tau-based neurodegenerative diseases.

The High-Indel CTE and AD individuals present a distinct mutational pattern. Using the counts of each context of PTA sIndels detected in each individual, unsupervised clustering identified High-Indel CTE and AD as a separate cluster from Low-Indel CTE and AD and neurotypical controls (Fig. 4C and fig. S12). The same clustering results were reinforced by META-CS data (fig. S13). The distinct pattern in High-Indel CTE and AD individuals is mainly characterized by 2 to 4 bp deletions, where the proportion of 2 to 4 bp deletions is significantly higher in High-Indel CTE compared to age-matched controls for both dsIndels and ssIndels (*P* = 1.0 × 10^−4^ and *P* = 7.5 × 10^−4^, two-tailed Wilcoxon test, Fig. 4D–E). In line with the fact that ID4 comprises primarily 2 to 4 bp deletions, we observed a consistent contribution of both double- and single-stranded ID4 across all High-Indel CTE (Fig. 4F), significantly higher than in Low-Indel CTE (fig. S14, dsIndel: *P* = 0.003, ssIndel: *P* = 0.005, two-tailed Wilcoxon test) and control groups (fig. S14, dsIndel: *P* = 0.001, ssIndel: *P* = 1.0 × 10^−4^, two-tailed Wilcoxon test). This highlights the role of ID4-associated biological processes in elevated DNA damage in CTE. Although the etiology of ID4 is not completely understood, recent studies (35, 36) demonstrated that ID4 may be mediated by topoisomerase 1 (TOP1) activity and linked to neurodegeneration.

## Influence of transcriptional activity and chromatin accessibility on somatic mutation in CTE

Gene transcription and epigenetic state are closely linked to DNA damage and repair in neurons (37, 38). In contrast to mitotic cells, replication-independent processes account for most somatic mutations in healthy neurons, where both gene expression and chromatin accessibility have been reported to be positively correlated with mutation burden (17, 18, 23). To investigate whether somatic mutations have distinct enrichment patterns in CTE neurons, we generated single-nucleus RNA sequencing (snRNA-seq) data from CTE and neurotypical control samples (fig. S15) and utilized published single-nucleus assay for transposase-accessible chromatin with sequencing (snATAC-seq) data from sample-matched neurotypical controls (23). We extracted neuronal profiles to eliminate potential biases from other cell types.

**Fig. 5.**
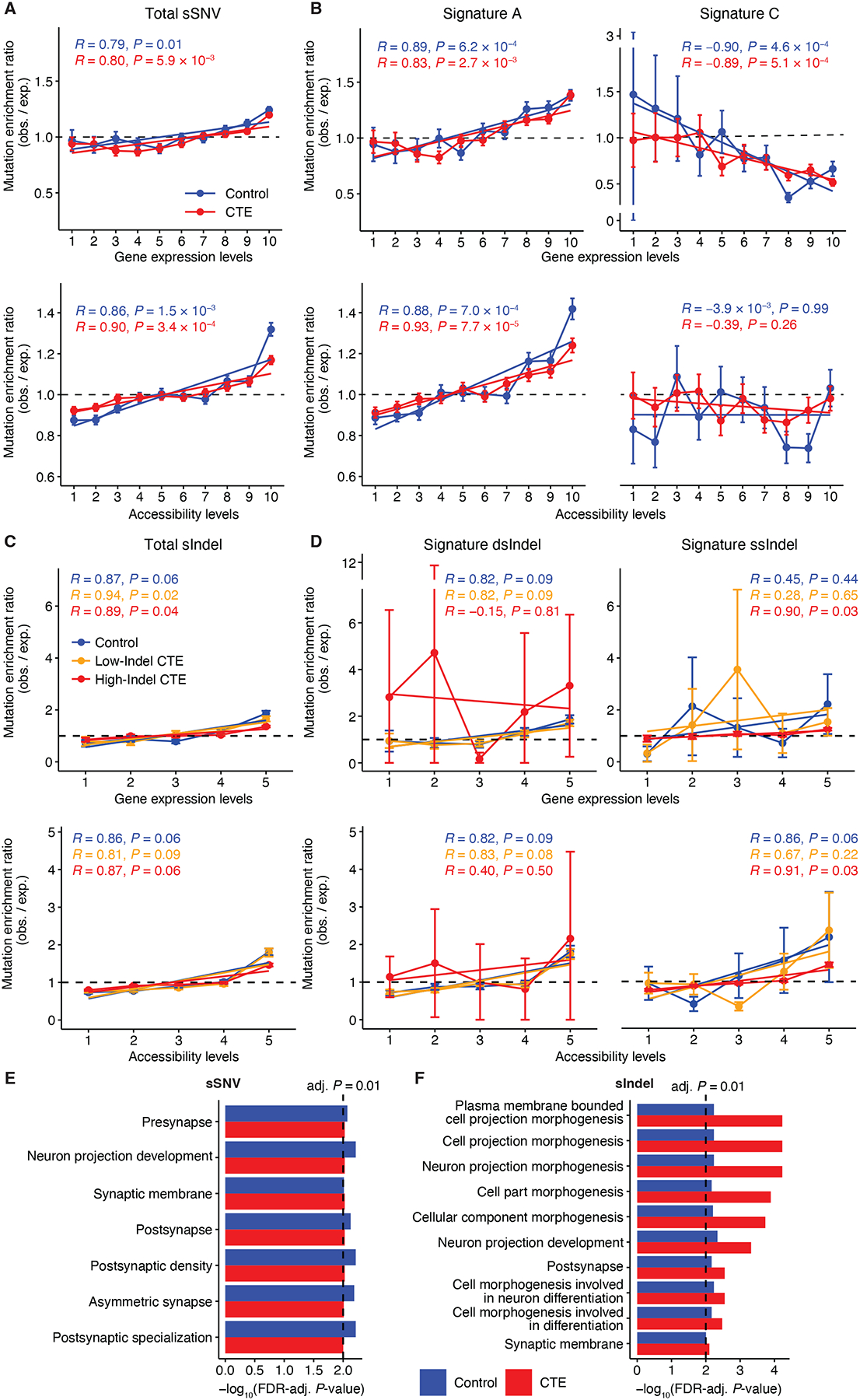
Enrichment analysis of sSNVs and sIndels in CTE neurons. (**A**-**D**) sSNV (A, B) and sIndel (C, D) density as a function of gene expression and chromatin accessibility levels. Neuronal transcriptional profiles were characterized from snRNA-seq data sequenced in this study. Neuronal accessibility profiles were obtained from snATAC-seq data in Ganz *et al*. (23). Genes and open chromatin regions are separated into 10 (for sSNV) or 5 (for sIndel) equally sized groups with increasing levels indicating increasing expression or accessibility. Observed density of each expression or accessibility group is obtained by overlapping the original mutation call sets with regions of each group. Expected density is calculated from 1000 permutations of mutation call sets overlapped with regions of each group. Enrichment ratio is calculated by observed / expected density for each permutation, and mean ratio over 1000 permutations is used to construct the trend line by linear model (error bar indicates standard deviation). Pearson correlation coefficient (*R*) and two-tailed p-value (*P*) are shown. obs.: observed, exp.: expected. (**E**, **F**) Gene ontology (GO) analysis of genes where sSNVs (E) and sIndels (F) are located. GO terms with FDR-adjusted *P* < 0.01 in both CTE and control are reported.

We found somatic mutation significantly more enriched in transcribed genes compared to non-transcribed genes in control and CTE (*P* < 0.05 for all, two-tailed Wilcoxon test), which aligns with previous findings and highlights the role of transcriptional activity in neuronal mutagenesis. We further confirmed positive correlations of somatic mutation with gene expression and chromatin accessibility in both control and CTE (Fig. 5A). Using signatures to further dissect enrichment patterns according to mutational processes, we observed positive correlations between gene expression and sSNVs attributed to Signature A for both control and CTE, while Signature C showed a negative correlation (Fig. 5B), consistent with previous results in AD neurons (18) and reinforcing the shared findings between CTE and AD. Chromatin accessibility showed no clear trend for Signature C-related sSNVs, which may be explained by complex relationships between chromatin accessibility and gene expression activity in highly expressed genes (fig. S16) as well as potential confounds of global genome (GG) NER-related sSNVs in regulatory elements, as Signature C was identified in genetic disorders with deficiency in either TC-NER or GG-NER (21). For sIndels, we separately extracted double- and single-stranded signatures from High-Indel CTE, Low-Indel CTE, and controls, and found that total burden as well as burden attributed to either signature showed moderate positive correlations with both gene expression and chromatin accessibility (Fig. 5C, D). The similar pattern between dsIndel and ssIndel suggests that they share similar mutagenic mechanisms, with single-stranded lesions representing an intermediate stage before double-stranded mutations.

We further examined the distribution of somatic mutations in protein-coding genes for their potential functional impact. Gene ontology (GO) analysis of genes mutated by sSNVs (Fig. 5E) and sIndels (Fig. 5F) revealed consistent enrichments in neuronal functions such as synaptic structures and neuron projection. This observation aligns with our finding that somatic mutations are enriched in regions of high expression and open chromatin, reflecting the active transcription of neuronal genes in neurons. Taken together with the CTE-specific somatic burden increase, our results suggest that excess somatic mutations, notably sIndels in CTE neurons, may frequently disrupt essential neuronal genes and eventually contribute to neurodegeneration (fig. S17).

## Discussion

In this study, we characterized somatic mutations in CTE neurons using two different scWGS methods and revealed elevated DNA damage leading to sSNVs and sIndels in CTE, in patterns distinct from normal aging. We propose specific mutagenic pathways for the accumulation of sSNVs and sIndels (fig. S17). Excess sSNVs in CTE were largely contributed by Signature C, a pattern that is rare in controls and elevated in AD. Given the shared feature of tau pathology, CTE and AD may share common pathways for accumulating DNA damage, possibly related to oxidative damage (18). We also found a subset of High-Indel CTE individuals harboring excess double- and single-stranded Indels, with a predominant contribution from ID4 (27). Analysis of AD individuals showed a subset with similar excessive sIndels, which was also seen in a parallel study examining tau pathologic states (39).

Previous studies have highlighted RHI as the likely cause of CTE (40, 41); however, not all individuals exposed to RHI develop CTE. We found that the somatic mutation burden of the RHI group was similar to neurotypical controls, implying that the elevated DNA damage in CTE is distinct from long-term exposure to RHI. A limitation of this study is that the RHI group is small with a narrow age range, and a larger group of varying ages and RHI exposures is needed to validate this finding. Our cohort focused on severe CTE (stage III and IV); future studies that include early-stage CTE could assess the role of somatic mutations in disease progression. Moreover, other unique features of CTE such as the perivascular localization of tau deposition at the depths of the cortical sulci may reveal novel associations between DNA damage and pathogenesis. Our analysis between the sulcus and gyrus showed no difference (fig. S4) but statistical power was limited. In addition, we did not detect any somatic mutations in genes known to be associated with neurodegenerative tauopathies (*APP*, *PSEN1*, *PSEN2*, *APOE*, and *MAPT*), similar to a previous report in AD (18).

Duplex sequencing data distinguished single-stranded and double-stranded genomic alterations, showing that sSNVs and sIndels in CTE neurons appeared to have distinct distributions of double- and single-stranded events that may be individual-specific. Most sSNVs from all CTE individuals were represented on both strands; however, High-Indel CTE individuals present a unique pattern contributed by many features of ID4. ID4 was recently reported to occur when ribonucleotides are mis-incorporated into DNA and subsequently cleaved by TOP1 instead of RNase H2 (35). These double-stranded deletions are thought to arise via an intermediate step of single-stranded > 1 bp deletions, which aligns with our observation that ID4-like patterns have a stronger signal in ssIndels. Although the association between RNase H2 deficiency and neurodegenerative disease is unknown, our results raise the possibility that a large quantity of genome-embedded ribonucleotides in CTE and AD neurons might overwhelm the ribonucleotide excision repair pathway, with the TOP1-mediated process leaving large numbers of > 1 bp deletions on single DNA strands of which only a small proportion could be efficiently repaired or fixed into double-stranded Indels in post-mitotic neurons (36). A parallel study (36) found a similar sIndel pattern in two other neurodegenerative diseases with distinct pathologies and provided direct experimental evidence of some expected features of TOP1-mediated mutagenesis, suggesting an even wider impact of TOP1 activity in neurodegeneration. It is also worth noting that ID4 may not fully explain the DNA damage in CTE, as some excess sIndels, particularly 2 bp deletions in one repeat unit, are limited in ID4. This suggests that other mutagenic processes may play a role in congruence with or independent of ID4-associated processes. Nonetheless, our analysis highlights that CTE can be viewed as a genomic process in affected neurons, with progressive transcription-related accumulation of sSNV and especially sIndels - which accumulate to numbers equivalent to > 100 years of excess aging - creating the potential for severe dysregulation of the neuronal transcriptome (fig. S17).

## Materials and Methods

### Data reporting

No statistical methods were used to predetermine sample size. Experimenters were not blinded, and experiments were not randomized.

### Human tissue samples and CTE case selection

Post-mortem frozen human brain tissues were obtained from the UNITE Brain Bank (formerly VA-BU-CLF) at Boston University, the Massachusetts Alzheimer’s Disease Research Center (MADRC) at Massachusetts General Hospital, and the NIH Neurobiobank at the University of Maryland Brain and Tissue Bank (UMBTB).

Tissue samples were collected and distributed for research and publication under the protocols approved by the Boston University-Veterans Affairs Institutional Review Board (for BU-VA: S07-02-0087), MassGeneral Brigham Institutional Review Boards (for MADRC: 1999P009556/MGH, expedited waiver category 5) and the University of Maryland Institutional Review Board (for UMBTB: 00042077), and after provision of written authorization and informed consent. Research on these de-identified specimens and data was performed at Boston Children’s Hospital with approval from the Committee on Clinical Investigation (S07-02-0087 with waiver of authorization, exempt category 4) and at Brigham and Women’s Hospital with approval from the MassGeneral Brigham Institutional Review Board (2019P003790 for secondary use as non-human subjects research).

Neurotypical control and AD cases were included from part of previous studies (17, 18, 23). Neurotypical control cases had no clinical history of neurological disorders, and Alzheimer’s disease cases were pathologically confirmed Braak stage V-VI. CTE cases were pathologically confirmed stage III-IV (3, 42). Neither AD nor CTE cases had other notable neurodegenerative pathology.

### Isolation of individual pyramidal neurons using FANS

Isolation of single neuronal nuclei was performed through fluorescence-activated nuclear sorting (FANS) for the neuronal nuclear transcription factor (NeuN), as described in previous work (18, 21, 43). In brief, nuclei were prepared from unfixed frozen (at −80 °C) human brain tissue in a dounce homogenizer using a chilled tissue lysis buffer (10 mM Tris-HCl, 0.32 M sucrose, 3 mM Mg(OAc)_2_, 5 mM CaCl_2_, 0.1 mM EDTA, 1 mM DTT, 0.1% Triton X-100, pH 8) on ice. Lysed tissue was then layered on top of a sucrose cushion buffer (1.8 M sucrose 3 mM Mg(OAc)_2_, 10 mM Tris-HCl, 1 mM DTT, pH 8) and ultra-centrifuged for 1 hour at 30,000 × g. Nuclear pellets were resuspended in ice-cold PBS supplemented with 3 mM MgCl_2_, filtered, then stained with DAPI and anti-NeuN (RBFOX3) antibody directly conjugated to Alexa Fluor 488 (AF488) (Millipore MAB377X, clone A60, 1:1,250; RRID: AB_2149209). DAPI staining allowed for isolation of single diploid neuronal nuclei apart from debris and doublet droplets. Previous work showed that using flow cytometry (with software BD FACSDiva v.8.0.2) to gate for the nuclei population with the highest NeuN signal as well as the highest forward scatter area (FSC-A) signal produced 99.3% excitatory pyramidal neurons (18).

### scWGS of pyramidal neurons using PTA

Isolated single nuclei were sorted one nucleus per well into 96-well plates and their genomes were amplified by PTA (22). The quasi-linear process of amplification in PTA has been found to reduce amplification artifacts and improve variant calling (17). PTA was performed using the ResolveDNA Whole Genome Amplification Kit (BioSkryb Genomics) as described in previous work (18). In brief, nuclei were sorted into 3 μl Cell Buffer pre-chilled on ice and lysed by addition of 3 μl MS Mix, with mixing at 1,400 rpm performed after each step. Lysed nuclei were then neutralized with 3 μl SN1 buffer, followed by 3 μl of SDX reagent, a 10-min incubation at room temperature, and 8 μl of reaction mix (containing polymerase) for a 20 μl of total reaction volume. Next, amplification was performed at 30 °C for 10 h, followed by enzyme inactivation at 65 °C for 3 min and DNA cleanup using AMPure. The yield was then determined by PicoGreen binding (Quant-iT dsDNA Assay Kit, Thermo Fisher Scientific). Multiplex PCR at four random genomic loci were used as quality control (21), where samples with positive amplification at all four loci were retained for Illumina sequencing.

Library preparation followed a modified KAPA HyperPlus Library Preparation protocol described in the ResolveDNA EA Whole Genome Amplification protocol. In brief, end repair and A-tailing were performed for 100–500 ng amplified DNA input, followed by adapter ligation using the SeqCap Adapter Kit (Roche, 07141548001) and cleanup using AMPure. After on-bead PCR amplification, size selection was carried out using AMPure for libraries with a size of 300–600 bp. Quality control was performed on selected libraries using PicoGreen and TapeStation HS DS100 Screen Tape (Agilent PN 5067–5584), followed by paired-end sequencing (150 bp × 2) on Illumina NovaSeq sequencers at 30× coverage (table S2). Data from PTA-amplified neuronal genomes in CTE and RHI were analyzed alongside previously reported data from control (17, 23) and AD neurons (18).

### scWGS of pyramidal neurons using modified META-CS

The genomes of single neuronal nuclei were amplified by a modified version of META-CS (19), a transposase-based whole genome amplification technique in which each DNA fragment is tagged and barcoded with 16 unique tags (Dataset S1 of Xing *et al*. (19)), allowing for single-cell, strand-resolved identification.

DNA oligos were ordered from IDT. Each of the 16 META-CS transposons were annealed and assembled into transposomes with Diagenode Tagmentase (Diagenode; C01070010) per manufacturer’s protocol and stored at −80 °C.

Single neuronal nuclei, isolated as previously described, were sorted one per well into 96 well plates, and lysed in 2 μl of 1x Single Cell Lysis Buffer (20 mM Tris, pH 8.0, 20 mM NaCl, 0.15% Triton X-100, 25 mM dithiothreitol, 1 mM EDTA, 1.5 mg/mL Thermolabile Proteinase K (TLPK) (NEB, P8111S)) at 30 °C for 1 h, 55 °C for 10 min. Single cell lysates were stored at −20 °C if not immediately amplified.

Lysed nuclei were then transposed by the addition of 8 μL transposition mix (5ul Diagenode 2X Tagmentation buffer (Diagenode; C01019043), 1 μl diluted META-CS transposome, 2 μl H_2_O), mixed at 1640 rpm for 1 min, spun down at 1500 rpm, and incubated at 55 °C for 15 min. Transposases were removed by the addition of 2 μL 6X stop buffer containing 300 mM NaCl, 45 mM EDTA, 0.01% Triton X-100, and 1 mg/mL TLPK, with mixing and incubation at 37 °C for 30 min, 55 °C for 10 min.

First-strand tagging was performed by the addition of 13 μL Strand Tagging Mix 1 containing 5 μL Q5 reaction buffer (NEB; B9027S), 5 μL Q5 high GC enhancer (NEB; B9028A), 0.85 μL 100 μM (total) Adp1 primer mix (Dataset S1 of Xing *et al*. (19)), 0.6 μL 100 mM MgCl_2_, 0.55 μL water, 0.5 μL 10 mM (each) dNTP mix (Thermo Scientific; R0192), 0.25 μL of 20 mg/mL bovine serum albumin (NEB; B9000S), and 0.25 μL Q5 DNA polymerase (NEB; M0491S), followed with mixing and incubation at 72 °C for 3 min, 98 °C for 30 s, 62 °C for 5 min, 72 °C for 1 min. ADP1 primers were removed with 1 μL Thermolabile Exonuclease I (NEB; M0568L), with mixing and incubation at 37 °C for 15 min, 65 °C for 5 min.

Second-strand tagging was performed by the addition of 4 μL Strand Tagging Mix 2 containing 1 μL Q5 reaction buffer, 1 μL Q5 high GC enhancer, 0.95 μL 100 μM (total) Adp2 primer mix (Dataset S1 of Xing *et al*. (19)), 0.85 μL water, 0.1 μL 10 mM (each) dNTP mix, and 0.1 μL Q5 DNA polymerase, and incubation at 72 °C for 3 min, 98 °C for 30 s, 62 °C for 5 min, 72 °C for 1 min. Adp2 primers were removed by repeating the exonuclease step described above.

Strand tagging products were amplified by the addition of 19 μL PCR mix containing 1 μL NEBNext Multiplex Oligos Universal Primer, 1 μL NEB Index Primers (NEB; E7335S, E7500S, E7710S, E7730S), 4 μL Q5 reaction buffer, 4 μL Q5 high GC enhancer, 0.4 μL 10 mM (each) dNTP mix, 8.4 μL water, and 0.2 μL Q5 DNA polymerase and incubation at 98 °C for 20 s, 13 cycles of [98 °C for 10 s, 72 °C for 2 min], 72 °C for 2 min.

Single-cell libraries were pooled together, and then libraries were purified by DNA Clean and Concentrator-5 columns (Zymo; D4013) and amplification efficiency was checked for fragment size and concentration by Agilent TapeStation or Agilent Bioanalyzer. Size selection was performed with Ampure XP beads (Beckman Coulter; A63880), wherein the pooled library was divided into three groups based on fragment size. Medium-size fragments (~300 – 1000 bp) were selected first by the addition of 0.6X beads then by a further addition of 0.25X beads (for a final 0.75X).

### snRNA-seq of CTE and control samples

Single-nucleus RNA sequencing (snRNA-seq) was performed on representative tissue samples (control individual UMB1465, prefrontal cortex; CTE individual CTE9130, prefrontal cortex) to assess mutational enrichment with gene expression. Isolation of nuclei was performed as described above with the following modifications: both 0.2 U μl^−1^ Protector RNAse inhibitor (Roche RNAINH-RO) and 0.2 U μl^−1^ SuPERase-IN RNAse inhibitor (Invitrogen) were added to the tissue lysis buffer and to the immunostaining buffer, and MgCl_2_ was omitted from the immunostaining buffer. For each sample, ~16,000 nuclei were sorted into one well of 96-well plates. snRNA-seq was performed using the 10X Genomics Next GEM Single Cell 3’ GEM Kit v3.1 and Chromium Controller, followed by Illumina sequencing.

### Read mapping and BAM file generation for bulk and PTA data

BWA (v0.7.15) (44) was first used to map reads from bulk WGS and PTA scWGS data onto the human reference genome (GRCh37 with decoy) with default parameters. Then, duplicate reads were marked by MarkDuplicates of Picard (v.2.8.0), followed by local Indel realignment and base quality score recalibration using Genome Analysis Toolkit (GATK) (v.3.5) (45) to generate BAM files for mutation calling.

### Quality measures of single-cell genome amplification

To evaluate the quality of single-cell genome amplification for both PTA and META-CS data, we used a number of measures. First, we calculated the median absolute pairwise differences (MAPD) to quantify the evenness of amplification as described previously (46). MAPD score was computed by binning the genome, estimating copy number of each bin, and taking the median of absolute pairwise differences between log2-transformed copy number ratios of adjacent bins. A lower MAPD score indicates more even amplification of the genome. Further, to account for the variance of the copy number ratio distribution, we calculated the coefficient of variation (CoV) of these absolute pairwise differences by taking the ratio of their standard deviation to their mean. Sequencing depth was estimated using the total number of properly mapped and paired reads (from samtools stats) multiplied by read length and divided by the whole genome length. To account for amplification bias, we estimated the allelic and locus dropout rates using a set of high-quality germline heterozygous SNPs that overlap with common variants from the 1000 Genome Project. Allelic dropout sites have either REF depth or ALT depth < 2. Locus dropout sites have total depth < 5. Strand dropout rate was estimated as the square root of allelic dropout rate.

### Calling of sSNVs and sIndels from PTA data

We used Single Cell ANalysis 2 (SCAN2, v.1.0) (17) to identify sSNVs and sIndels from single-cell PTA data with matched bulk data. First, we generated four cross-sample panels. One panel for 17 control individuals from UMBTB, one panel for 4 RHI individuals, one panel for 15 CTE individuals and 2 control individuals from UNITE Brain Bank (formerly VA-BU-CLF), and one panel for 7 AD individuals. Each panel was configurated by running “scan2 config --analysis makepanel” with following reference parameters, human reference genome GRCh37 with decoy (--ref), dbSNP v138 common variants (--dbsnp), and 1000 Genomes Phase 3 SHAPEIT2 phasing panel (--shapeit-refpanel). Each panel was then built by running “scan2 makepanel”. After panel generation, mutation calling was performed for each individual by running “scan2 config -- analysis call_mutations” with the same reference parameters above, each PTA BAM (--sc-bam), matched bulk BAM (--bulk-bam), and the corresponding cross-sample panel (--cross-sample-panel), followed by “scan2 run”. For each single cell, SCAN2 generated both mutation calls and genome-wide somatic mutation burden estimations (autosomes only) which were used for the subsequent PTA analyses described below. Two AD cells were excluded from this study due to either failed SCAN2 run (ALZ1647BA9-C) or large discrepancy in burden estimations between SCAN2 and LiRA (1995P_201001E3), the tool originally used to profile these cells (18).

### Preprocessing and filtering of META-CS data

Our pipeline to preprocess META-CS data and perform sSNV calling shares a core workflow with the previously reported method (19) and includes extra steps to further strengthen accuracy and remove false positives which better accommodates our modified experimental protocol. First, paired-end reads were preprocessed by pre-meta including identifying transposon barcodes, merging overlapping read ends, and trimming Illumina adapters. Then, two aligners, BWA-MEM (v.0.7.17) (47) and Minimap2 (v.2.12) (48), were used to map reads to the human reference genome (GRCh37 with decoy) and generate BAM files. Of note, since each original DNA fragment was tagged by a pair of transposon barcodes, we split BAM files by barcode pairs before running mutation calling in each barcode pair BAM. This allows us to filter out reads without matching barcodes and ensure that calling was performed on a single-molecule level (i.e. for each allele). In addition, given the relatively small number of unique barcodes, there is a chance of barcode collision where different DNA fragments are tagged by the same barcode pair. Therefore, we extracted Tn5 cut sites from each read pair, with the assumption that reads amplified from the same DNA fragment should share the same Tn5 cut sites. Other quality metrics were obtained in the same way as PTA data (described above). Due to batch effects and imbalanced coverage among pooled cells, cells with an average insert size < 280 or > 500, or a standard deviation of insert size > 750 were filtered out. In addition, some cells had abnormally high numbers of calls, and we found a significant overlap (~93%) between their pre-filtered call sets and the gnomAD (49) SNPs of ≥ 1% population frequency. These cells were removed due to potentially contamination with another individual.

### Calling of sSNVs from META-CS data

We generated sSNV candidates by identifying variants that have no non-reference (ALT) allele read from bulk but at least four total ALT reads and at least two ALT reads from each strand for duplex support in the cell. To achieve a robust and accurate calling, we filtered out candidate sites that satisfied any of the following criteria: overlapping with the low-quality regions as previously reported (19), overlapping with gnomAD (49) SNPs of ≥ 1% population frequency, within 100 bp from another candidate site. Passing variants need to have at least 4 ALT reads in total with at least 2 ALT reads from each strand (a4s2) as well as a VAF = 1 at single-molecule level.

### Calling of sIndels from META-CS data

We established a new pipeline (https://github.com/gldong/duplex-indel) for sIndel calling from META-CS data by introducing novel modules to expand on the sSNV pipeline (manuscript in preparation). First, as the majority of false positive calls originated from incorrectly merged read pairs during preprocessing, we created a module to tag the merged reads and calculate a genomic window where the merging occurred. Second, we generated an additional BAM file without read merging and filtered out any candidate sites that either overlapped with the read merging window or were not present in the BAM without read merging. In addition, we removed any candidate sites that were adjacent to a germline Indel (i.e. located within the flanking region of the germline Indel start site; flanking region is defined by 5 bp or twice of the germline Indel length on both sides, whichever is larger). Other filtering criteria were the same as the ones used for sSNV calling, except that we used gnomAD (49) Indels of ≥ 1% population frequency. Passing variants need to have at least 4 ALT reads in total with at least 2 ALT reads from each strand (a4s2) as well as a VAF = 1 at single-molecule level.

### Calling of single-stranded variants from META-CS data

Single-stranded SNV and Indel calls were detected in a similar fashion as double-stranded calls described above. We generated candidate sites by identifying variants that have no ALT read from bulk, at least four ALT reads from the variant strand in the cell, and no ALT read from the non-variant strand in the cell. Then, the same filtering strategy except for the ALT allele balance was implemented to generate the final call set. Passing variants need to have at least 4 ALT reads from the variant strand and at least 4 REF reads from the non-variant strand.

### Additional filtering of SNV and Indel calls from META-CS

To account for batch effects and technical artifacts, we implemented a set of filters to remove false positives. The same filters were applied to both double- and single-stranded SNVs and Indels. We removed variant sites that meet at least one of the following criteria: 1) variant site is covered by more than one barcode-pair, 2) barcode-pair covering the variant site has more than one set of Tn5 cut sites, 3) variant site located within 20 bp of read ends.

### Statistical models of somatic mutation burden

We used linear mixed-effects (LME) models from the lme4 (v.1.1–27.1) R package (50) to investigate potential associations between somatic mutation burden and other covariates of interest. Somatic mutation burden was modeled as a continuous outcome, covariates of interest including age, clinical status, and cell quality measures were modeled as fixed effects, and individuals were modeled as random effects due to potential correlations between cells from the same individual. P-values of fixed effects were obtained from t-tests with the Satterthwaite approximation using the lmerTest (v.3.1–3) R package (51). Each clinical group was tested against controls separately to obtain the corresponding p-value. To calculate QC-corrected burden, we first modeled each quality measure and age as fixed effects in control neurons to obtain the coefficient of this quality measure’s contribution, and then subtracted its contribution from total somatic burden of each cell before modeling the corrected somatic burden against age and clinical status to generate QC-corrected burden comparisons.

As disease cells often exhibit large variation in somatic mutation burden (which could also represent biological effects), the various assumptions of the LME model may not always hold. Therefore, to strengthen our statistical testing results, we performed additional non-parametric Wilcoxon tests on excess mutation burdens across clinical groups after adjusting for age. To calculate the age effects on mutation burden, we fitted burdens from control cells in an LME model with age as fixed effects and individuals as random effects, and checked the model assumptions (linearity, homoscedasticity, and normality) (fig. S2). Then, we used the coefficient of age to calculate an expected age-contributed mutation burden in cells across clinical groups, before subtracting it from the cell’s total mutation burden to obtain excess mutation burden. Finally, we used two-tailed Wilcoxon test, a non-parametric test that does not require those assumptions for LME, to test the difference in excess mutation burdens between pairs of clinical groups.

### Association analysis of somatic mutation burden

We used the same LME model described above to evaluate the association between somatic mutation burden and covariates of interest in CTE individuals. For years of playing football, sSNV or sIndel burden was modeled as a continuous outcome, age and the number of years playing football were modeled as fixed effects, and individuals were modeled as random effects. P-values of fixed effects were obtained from t-tests with the Satterthwaite approximation using the lmerTest (v.3.1–3) R package (51). For *APOE* ε4, we first genotyped all CTE individuals at two SNPs rs429358 and rs7412 using their bulk WGS data (6 possible genotypes are ε2/ε2, ε2/ε3, ε2/ε4, ε3/ε3, ε3/ε4, and ε4/ε4). Then, we used three genetic models of ε4 allele, dominant, additive (with linear penetrance), and recessive, to test the association between *APOE* ε4 genotype and somatic burden. Somatic burden was modeled as a continuous outcome, age and *APOE* ε4 genotype were modeled as fixed effects, and individuals were modeled as random effects. P-values of fixed effects were obtained from t-tests with the Satterthwaite approximation using the lmerTest (v.3.1–3) R package (51).

### Double- and single-stranded signature analysis

Strand-specific signatures were extracted from META-CS call sets and normalized. To better interpret these signatures, we decomposed them to the COSMIC v3.2 database (https://cancer.sanger.ac.uk/signatures) with matched mutation type using MutationalPatterns (v.3.0.1) R package (30).

Since these signatures were derived independently, the double- and single-stranded signatures for each mutation type exhibited collinearity that made them difficult for refitting. Therefore, two intermediate signatures were introduced to serve as a proxy for refitting. For each mutation type, the double- and single-stranded signatures, DS and SS, were combined and normalized, from which two intermediate signatures, IM_1_ and IM_2_, were extracted using NMF. DS and SS were then refitted to IM_1_ and IM_2_ so that they could be reconstructed as

$$\hat{DS}=a_{1}\times IM_{1}+a_{2}\times IM_{2},$$

$$\hat{SS}=b_{1}\times IM_{1}+b_{2}\times IM_{2},$$

where a_1_, a_2_, b_1_, and b_2_ were proportional contributions normalized to IM_1_ and IM_2_ (i.e. a_1_ + b_1_ = 1 and a_2_ + b_2_ = 1).

To estimate the proportions of double- and single-stranded calls within the PTA call sets, for each mutation type in each cell, PTA calls were fit to the intermediate signatures,

$$\hat{PTA}=c_{1}\times IM_{1}+c_{2}\times IM_{2},$$

and the signature contributions were converted to describe DS and SS,

$$\text{PT}\hat{A_{\text{converted}}}=k_{1}\times DS+k_{2}\times SS,$$

where *k*_1_ = *c*_1_ × *a*_1_ + *c*_2_ × *a*_2_ and *k*_2_ = *c*_1_ × *b*_1_ + *c*_2_ × *b*_2_. Note that all signatures and spectra are vectors.

### Known and *de novo* mutational signature analysis

To study the contributions of known and *de novo* mutational signatures to the PTA call sets, we first categorized the mutation calls from each cell into context groups predefined by COSMIC v3.2 (96 contexts for SNVs and 83 contexts for Indels). For sSNVs, we used MutationalPatterns (30) to fit the PTA calls to known signatures A and C reported in Lodato *et al*. (21) to get the proportional contribution of each signature to each cell. Then, signature-specific somatic mutation burden was calculated by multiplying the contribution by the genome-wide burden, which is comparable between cells. *De novo* signatures were extracted using the NMF-based method in MutationalPatterns. The number of signatures to extract was determined with 200 NMF runs based on the Kullback-Leibler divergence (method = “brunet”). PTA calls were fitted to the *de novo* signatures in the same way as for known signatures. We used the COSMIC v3.2 database to decompose both known and *de novo* signatures for further interpretation.

### Permutation of sSNV and sIndel calls

Permutation sets are crucial for robust enrichment analyses by controlling for potential biases that exist in the original mutation calls and providing powered statistical tests to evaluate the significance of enrichment. For each permutation, the original calls were randomly shuffled across the callable regions of each cell while preserving their chromosome and trinucleotide context. We generated 1000 permutation sets for each mutation type in each cell using SCAN2 by running “scan2 config --analysis permtool” with original mutation calls (--permtool-muts), the human reference genome GRCh37 with decoy (--permtool-bedtools-genome-file), and 1000 permutations (--permtool-n-permutations), followed by “scan2 permtool”.

### Droplet-based snRNA-seq analysis

Gene count matrices for both control and CTE were acquired by aligning reads to the GRCh37 genome (v.3.0.0) using Cellranger (v.6.1.0) (52) with default parameters. Then we used the standard workflow from Seurat (v.4.0.5) (53) to process the snRNA-seq data. For the purpose of quality control, we removed genes that were not expressed in < 3 cells and cells with 1) < 200 genes, 2) < 500 counts or > 15000 counts, or 3) mitochondrial gene percentage > 5%. After applying these filters, we obtained 4619 cells for the control dataset and 4080 cells for the CTE dataset. Next, the data were normalized using the function “LogNormalize” with “scale.factor” = 10000 and scaled to a maximum value of 10. We then performed dimension reduction (principal component analysis, t-SNE, and UMAP) and cell clustering using Louvain (54). To identify the cell types that were captured in the snRNA-seq data, we selected marker genes for each cluster by statistical tests and assigned cell-type labels according to previously reported marker lists (55). Through visual evaluation, cells with the same identity were clustered together.

### Enrichment analysis with snRNA-seq and snATAC-seq data

We performed enrichment analysis of somatic mutations in transcribed gene or open chromatin regions using our in-house snRNA-seq data and previously reported snATAC-seq data generated from matching control samples (23). We extracted the gene expression and chromatin accessibility profiles of neurons annotated in snRNA-seq and snATAC-seq data to match the PTA data.

For gene expression enrichment analysis, we used the expression profile of control for mutation calls from control and RHI cells, and the expression profile of CTE for mutation calls from CTE cells. First, genes were ranked based on their expression and divided into equal-sized groups (10 groups for sSNVs, 5 groups for sIndels). Then, we intersected both original and permuted mutation calls with gene regions of each expression group. Gene regions were annotated by ANNOVAR (56) using the database GRCh37 refGene (57). Mutation calls that overlapped with multiple genes were removed. A ratio of observed to expected number of calls (i.e., enrichment ratio) was calculated for each permutation round where the observed number came from the original call set and the expected number came from the permuted call set. Enrichment was reported as the mean and standard deviation of the 1000 ratios. To test enrichment differences between transcribed and non-transcribed genes, we defined genes with an average expression > 0 as transcribed and the remaining genes as non-transcribed in either control or CTE sample after QC and normalization described above. We counted mutation numbers in transcribed vs. non-transcribed genes for each permutation and divided the observed counts over permuted counts to calculate an enrichment ratio for each transcriptional status in each clinical group. Then, a two-tailed Wilcoxon test was performed between transcribed and non-transcribed enrichment ratios in each group.

For chromatin accessibility enrichment analysis, we used the processed BED file of excitatory neurons (23) where the genome was first separated into non-overlapping 1000 bp bins and snATAC-seq fragments were mapped to the bins. Similar to gene expression enrichment, the bins were ranked based on their coverage and divided into equal-sized groups (10 groups for sSNVs, 5 groups for sIndels). Then, we intersected both original and permuted mutation calls with genomic regions of each accessibility group. Enrichment ratios were calculated in the same way as above.

In addition to total somatic mutation enrichment, we performed signature-specific enrichment using the same NMF-based method for signature analysis above. For both original and permuted calls that overlapped with each expression or accessibility group, we fit the sSNV calls to Signatures A and C and the sIndel calls to the double- and single-stranded signatures from META-CS. The observed/expected ratios were calculated as above.

### Gene Ontology analysis

Gene Ontology enrichment analysis was performed on genes that overlapped with somatic mutations using GOseq (v.1.42.0) (58) after controlling for gene length bias. A null distribution was generated by Wallenius approximation, and GO categories with less than 10 hits or more than 1000 genes were filtered out. P-values of over- and under-represented GO categories were adjusted for multiple testing using FDR. All GO categories with adjusted *P* < 0.05 in both CTE and neurotypical controls were reported in table S7.

## Supplementary Material

Supplementary Figures and Table S1

Supplementary Table S4

Supplementary Table S3

Supplementary Table S2

Supplementary Table S7

Supplementary Table S5

Supplementary Table S6

Figs. S1 to S17

Tables S1 to S7

References (42–58)

## Data Availability

Sequencing data generated in this study have been deposited in the NIAGADS repository under accession number NG00183 with controlled use conditions set by human privacy regulations. Previously published PTA data for neurotypical control can be downloaded from dbGaP (accession number phs001485.v3.p1), and PTA data for AD can be downloaded from NIAGADS (accession number NG00121). All the code used in this study is publicly available at https://zenodo.org/records/15300743.
