# Supplementary material for: Diverse somatic genomic alterations in single neurons in chronic traumatic encephalopathy
Source: Science. Author manuscript; Available in PMC 2025 Nov 8. (PMC12594281; doi:10.1126/science.adu1351)
Supplement: Supplementary Figures and Table S1 [file NIHMS2118895-supplement-Supplementary_Figures_and_Table_S1.pdf]

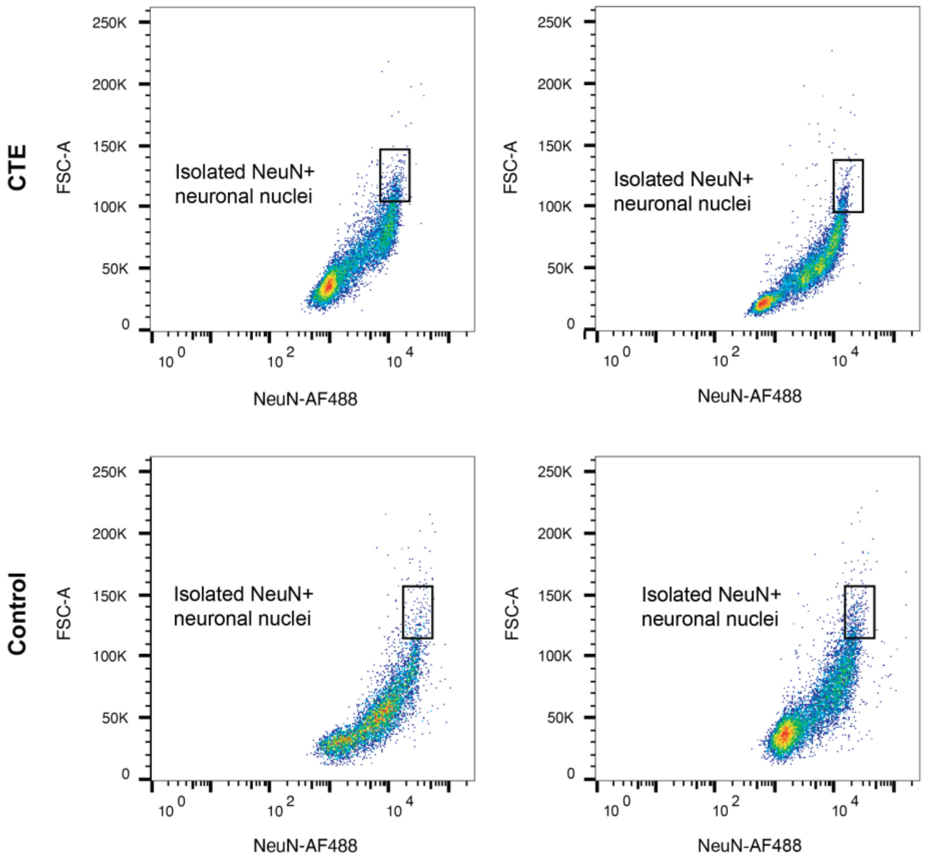

Fig. S1. Fluorescence-activated nuclear sorting (FANS) to isolate neurons. Staining with 4′,6-diamidino-2-phenylindole (DAPI) is used to identify intact diploid cellular nuclei, from which large (high FSC-A) neuronal nuclei are isolated using an AF488-conjugated anti-NeuN antibody. FANS plots for representative CTE (*left*: 7038; *right*: 9130) and control (*left*: 936; *right*: 5823) cases are shown. The FSC-A versus NeuN event distributions for diploid nuclei are similar across cases and clinical conditions. The black gate (rectangle) represents the subset of high-NeuN and high-FSC-A neurons that were sorted for single-cell whole-genome sequencing in this study. The color gradient represents the event density: high density in red, mid-range density in yellow, mid-low density in green, and low density in blue. Nuclei isolated in this manner represent > 99% pyramidal excitatory neurons (*18*).

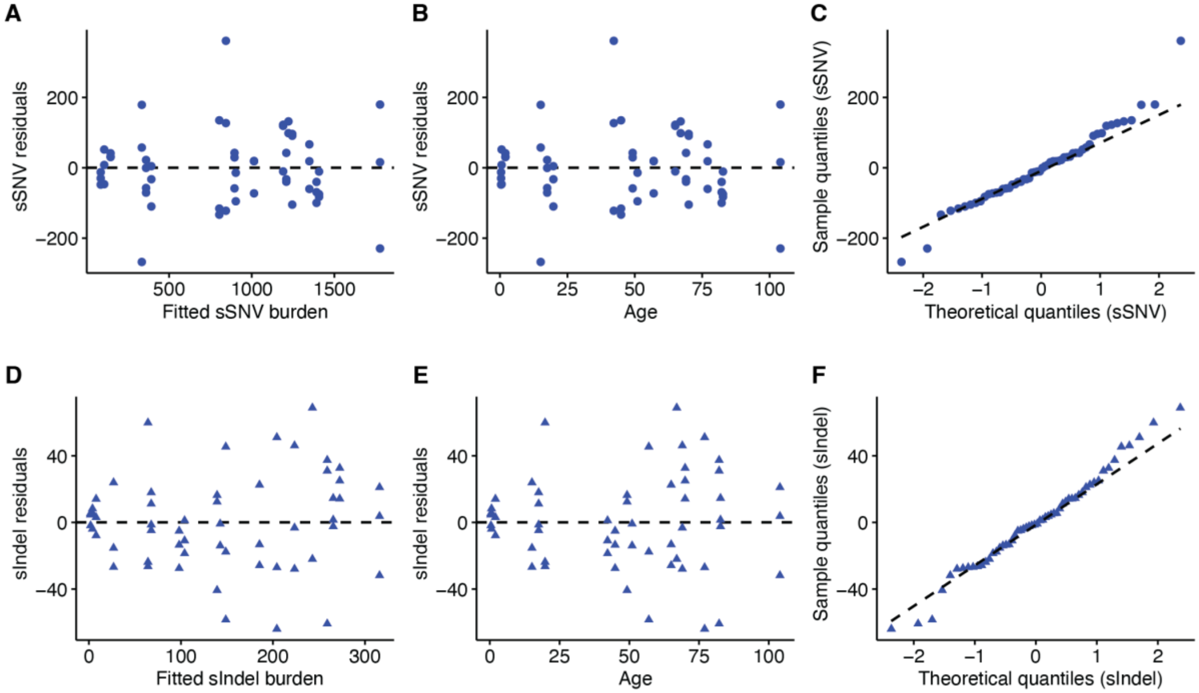

Fig. S2. Residuals of somatic mutation burden from fitted LME models in neurotypical control neurons. sSNV (**A-C**) and sIndel (**D-F**) burdens of neurotypical control neurons were fitted in LME models with age as fixed effects and individuals as random effects (see Methods). Residuals from fitted models are plotted against fitted burden (A, D) and age (B, E), and in Q-Q plots (C, F) to assess whether model assumptions including linearity, homoscedasticity, and normality are satisfied.

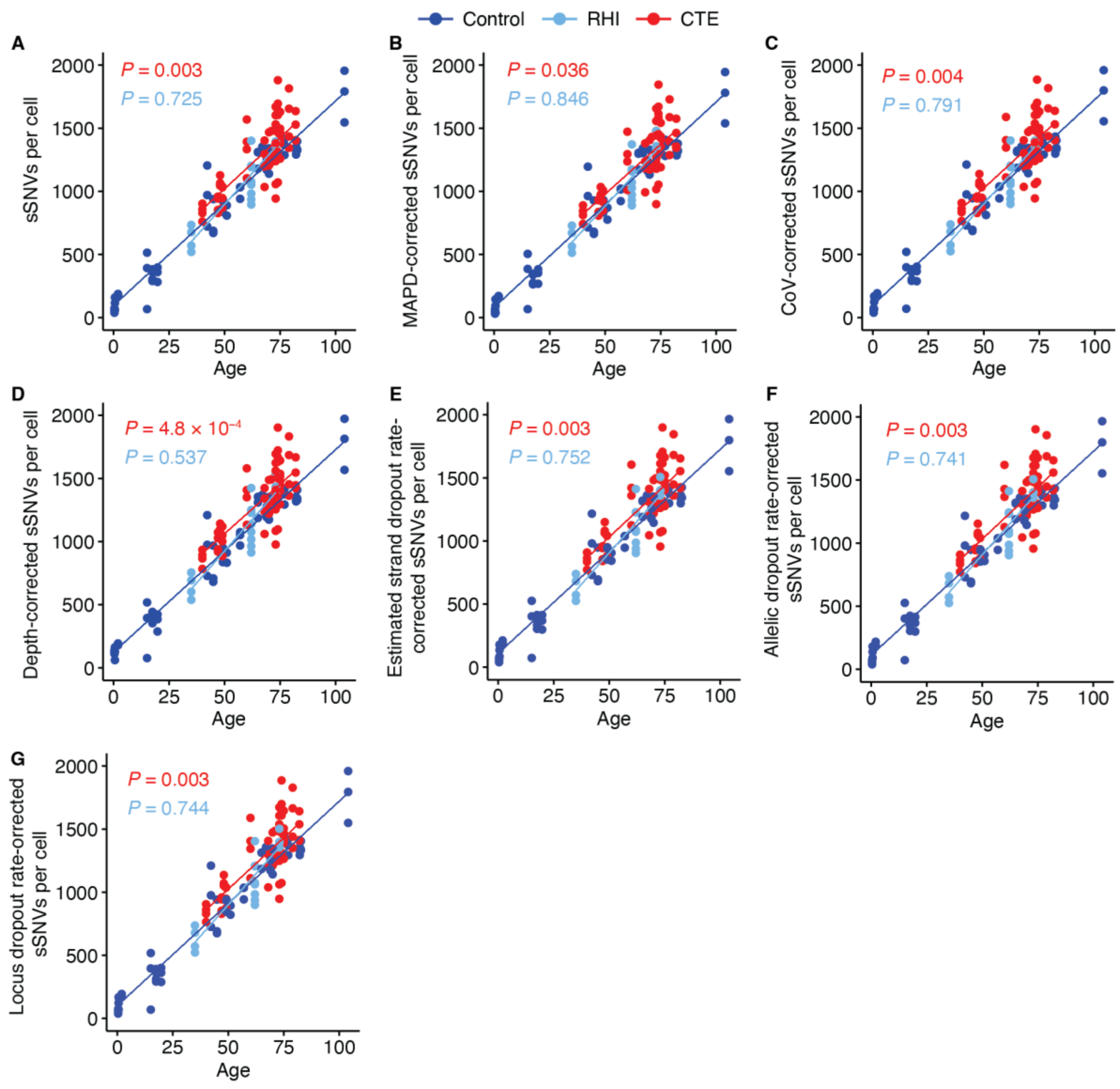

Fig. S3. sSNV burden in CTE (red), RHI (light blue), and neurotypical control (dark blue) neurons after controlling for QC metrics. To evaluate the potential confounding effect of sample and sequencing quality on unadjusted sSNV burden (**A**, reproduced from Fig. 1C), we calculated the sSNV burden corrected for five metrics (**B**–**G**). After controlling for MAPD (B), CoV (C), sequencing depth (D), estimated strand dropout rate (estimated as the square root of allelic dropout rate) (E), allelic dropout rate (F), and locus dropout rate (G), sSNV burdens in CTE and RHI are compared to neurotypical controls. P-values compare each clinical condition against controls.

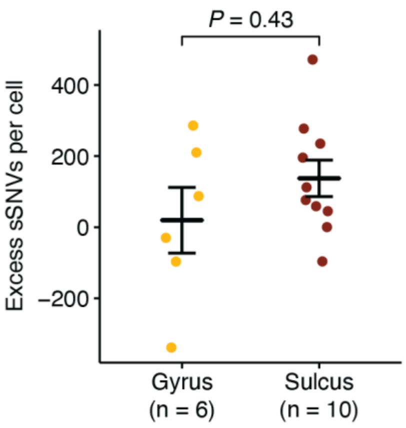

Fig. S4. sSNV burden comparison between neurons isolated from gyrus and from sulcus. sSNV burdens of neurons isolated from gyrus and from sulcus in CTE are compared using two-tailed Wilcoxon test. Each point is a single neuron. There are three CTE cases with neurons isolated from both loci, restricting the statistical power. Data are mean ± standard error.

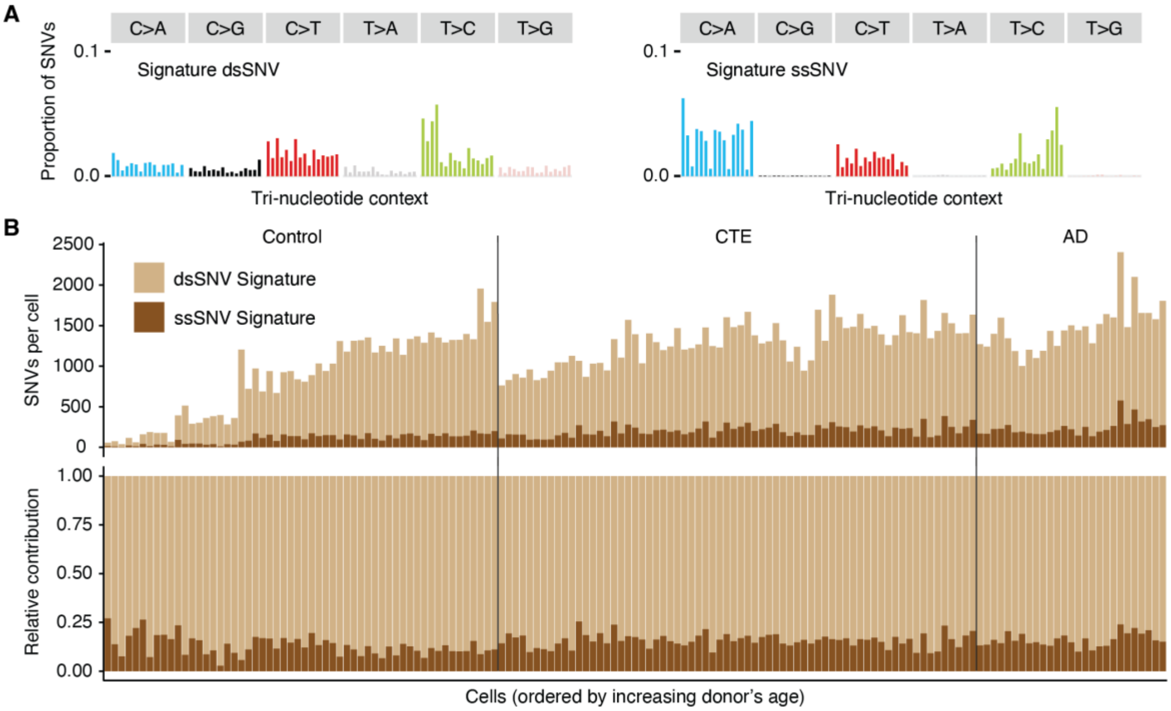

Fig. S5. Double-stranded (ds) and single-stranded (ss) SNV signatures extracted from META-CS data and their contribution in PTA neurons across clinical conditions. (**A**) dsSNV and ssSNV signatures are extracted from respective call sets of META-CS data. (**B**) Absolute (*top*) and relative (*bottom*) contribution of dsSNV and ssSNV signatures in PTA-profiled neurotypical control, CTE, and AD.

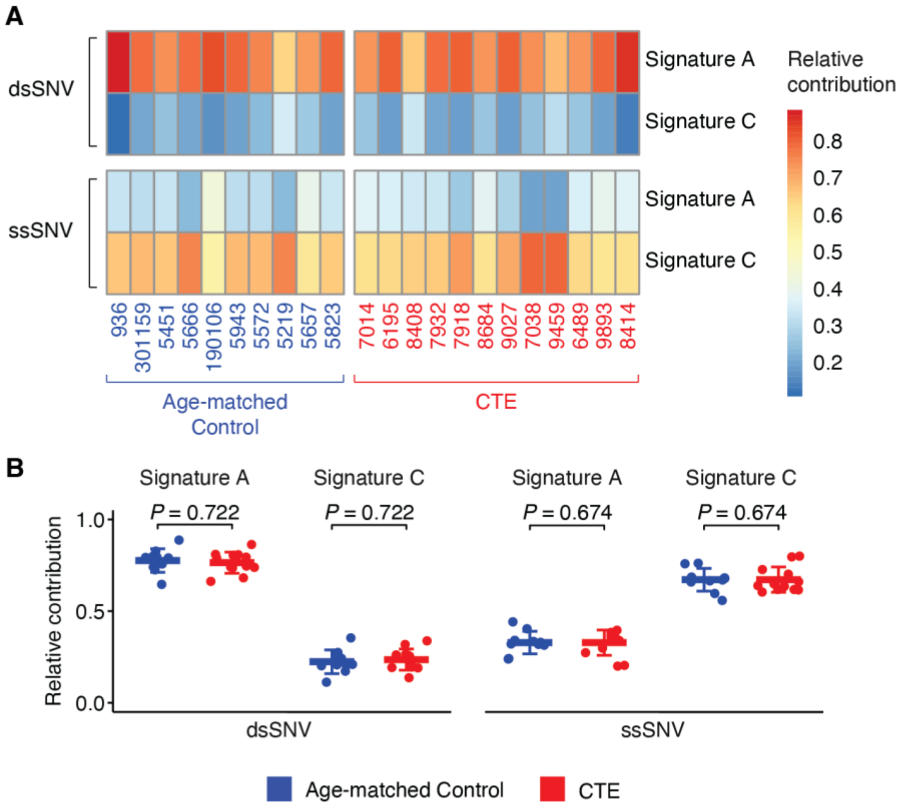

Fig. S6. Contribution of Signatures A and C in double-stranded and single-stranded SNVs. (**A**) After decomposing dsSNVs and ssSNVs into Signatures A and C for each case using META-CS data, relative contributions of the two signatures are shown as a heatmap. Case IDs are colored by their group assignment (age-matched control: dark blue, CTE: red). (**B**) Comparison of relative contribution of Signatures A and C to dsSNVs *(left)* and ssSNVs *(right)* between age-matched controls and CTE. Each point represents an individual from META-CS. Data are mean ± standard deviation. P-values are from two-tailed Wilcoxon tests.

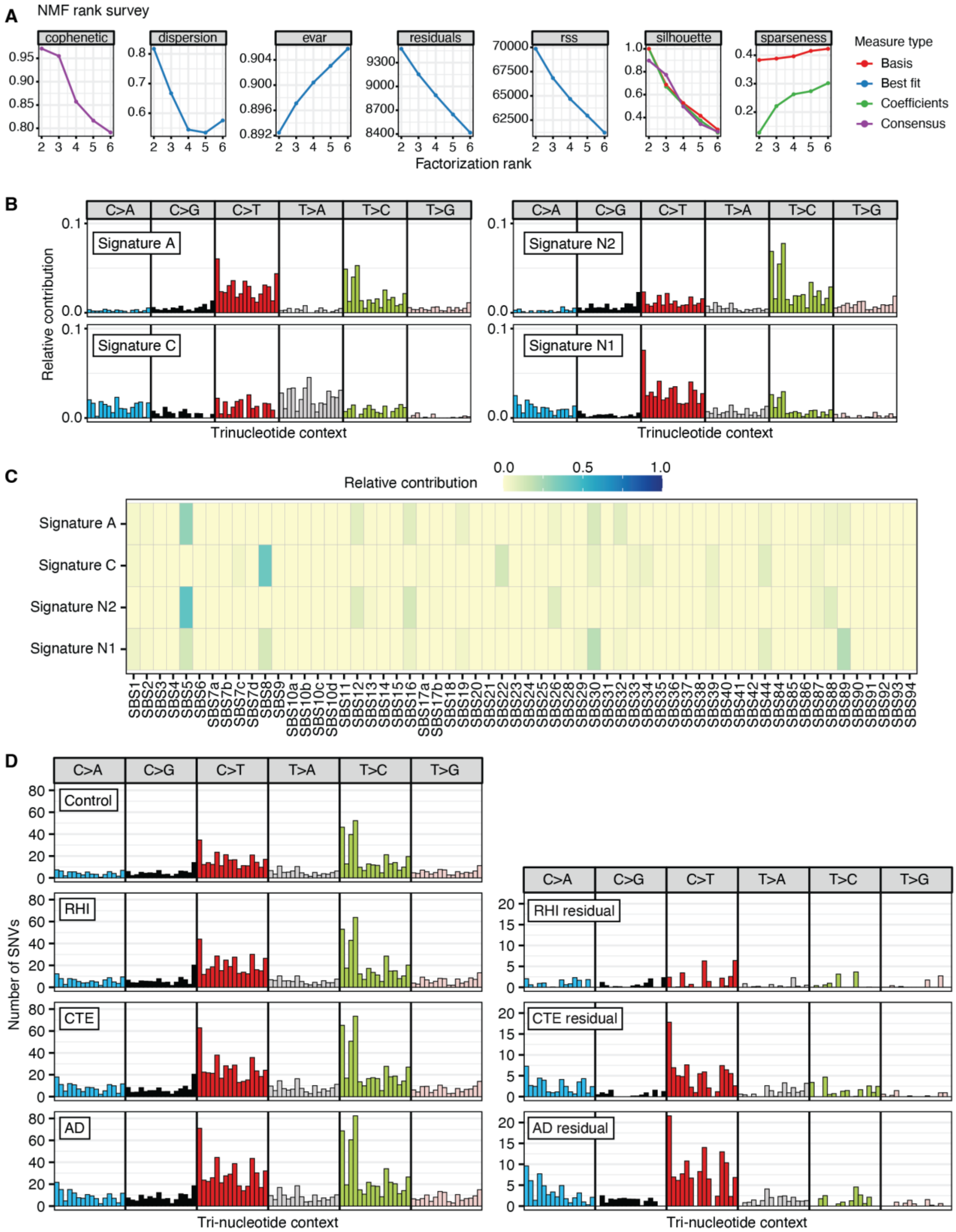

Fig. S7. *De novo* signature analysis and mutational patterns of sSNVs. (**A**) The optimal number of signatures to extract from PTA data is determined by NMF rank survey after 200 runs and specifically based on the cophenetic coefficient. (**B**) Mutational spectra of two *de novo*

signatures N1 and N2 which show a broad resemblance to Signatures C and A, respectively. (**C**) Decomposition of signatures A, C, N1, N2 to COSMIC SBS database. (**D**) Mutational patterns of PTA call sets in neurotypical controls, RHI, CTE, and AD (*left*). After subtracting the pattern of age-matched controls from RHI, CTE, and AD, residual patterns (*right*) are more specific to each clinical condition.

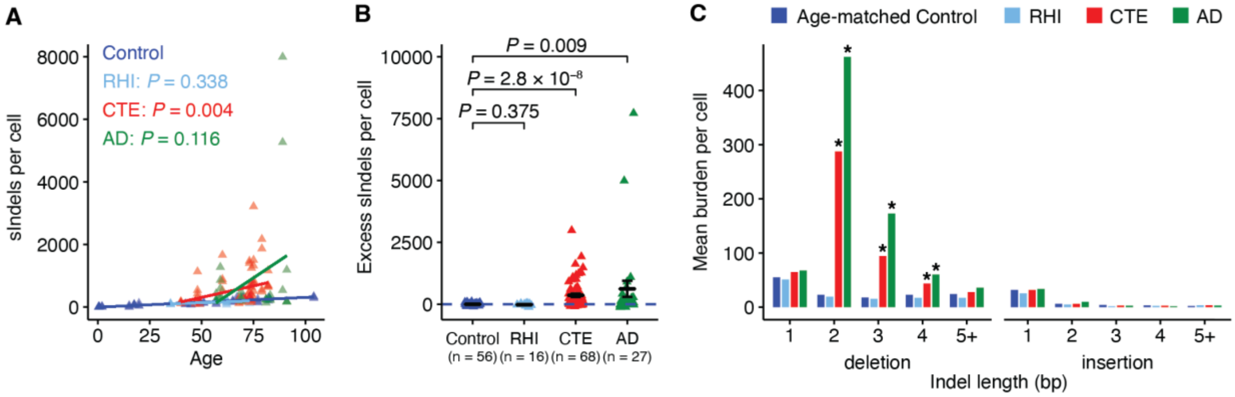

Fig. S8. sIndel burden and patterns in CTE and AD neurons. (**A**) sIndel burden in CTE (red), AD (green), RHI (light blue), and neurotypical control brains (dark blue). sIndel burden (from each neuron as a triangle) estimated by SCAN2 is fitted against age by clinical conditions using LME models (neurotypical control: dark blue; RHI: light blue, $P = 0.338$; CTE: red, $P = 0.004$; AD: green, $P = 0.116$). P-values compare each clinical condition against controls. (**B**) Excess sIndel burden in RHI, CTE, and AD compared to neurotypical control after adjusting for age. Data are mean ± standard error. The dashed blue line shows sIndels attributable to age (zero excess). P-values are from two-tailed Wilcoxon tests. (**C**) Comparison of all types of sIndels across age-matched controls, RHI, CTE, and AD. Data are mean burden per cell. Asterisk denotes significant changes in certain types of sIndels when compared to age-matched controls ($P < 0.05$, two-tailed Wilcoxon test).

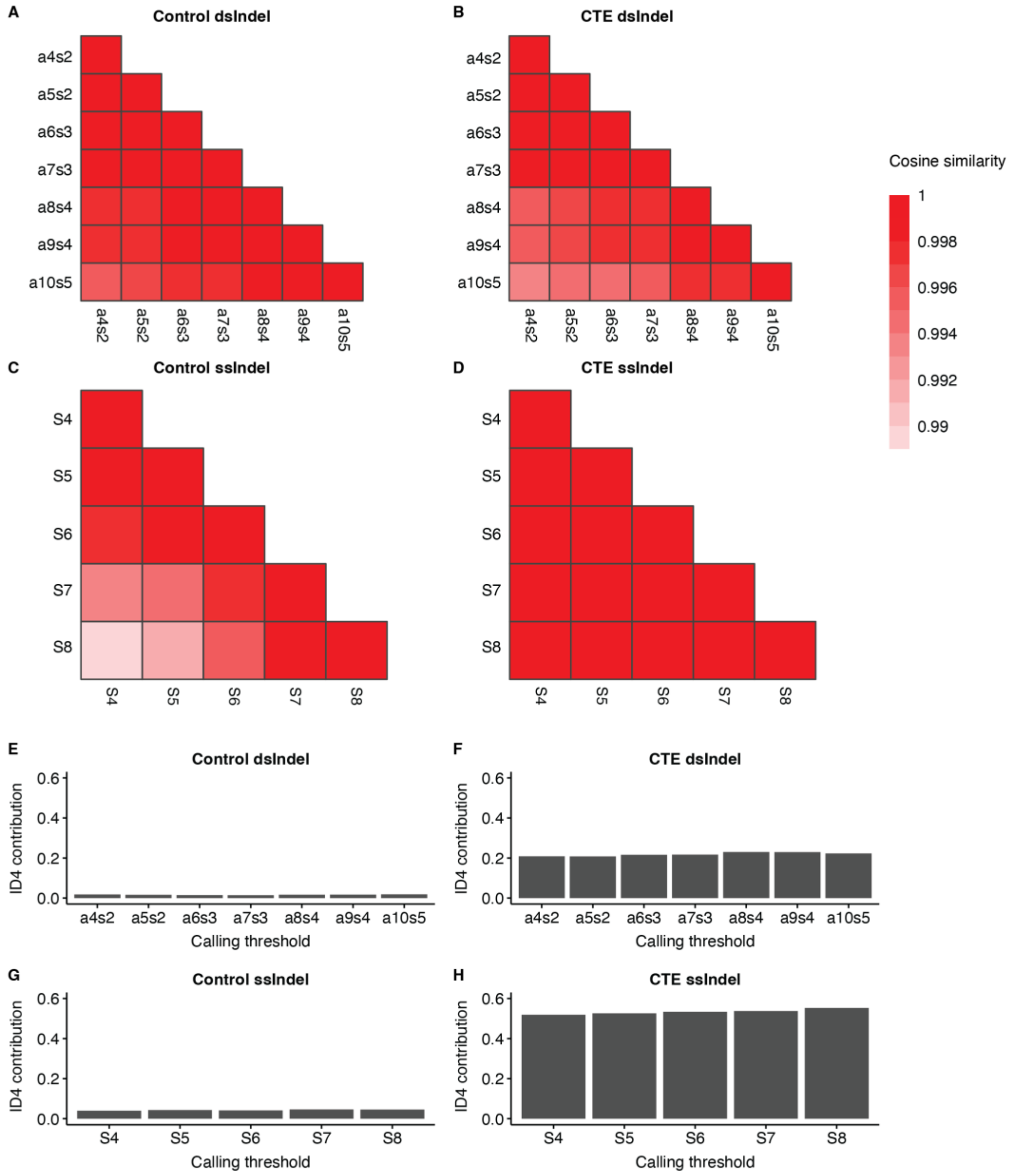

Fig. S9. Robustness of META-CS dsIndel and ssIndel signatures and ID4 contribution. (**A–D**) Cosine similarity across dsIndel and ssIndel signatures of neurotypical controls and CTE extracted from call sets based on varying calling thresholds. (A, B) Current calling threshold for a dsIndel site is at least 4 total non-reference reads (a4) with at least 2 non-reference reads from each strand (s2). (C, D) Current calling threshold for a ssIndel site is at least 4 reads from each strand (S4) with reference allele on one strand and alternative allele on the other strand. (**E–H**) ID4 contribution based on COSMIC decomposition of dsIndel and ssIndel signatures from varying calling thresholds shown in A-D.

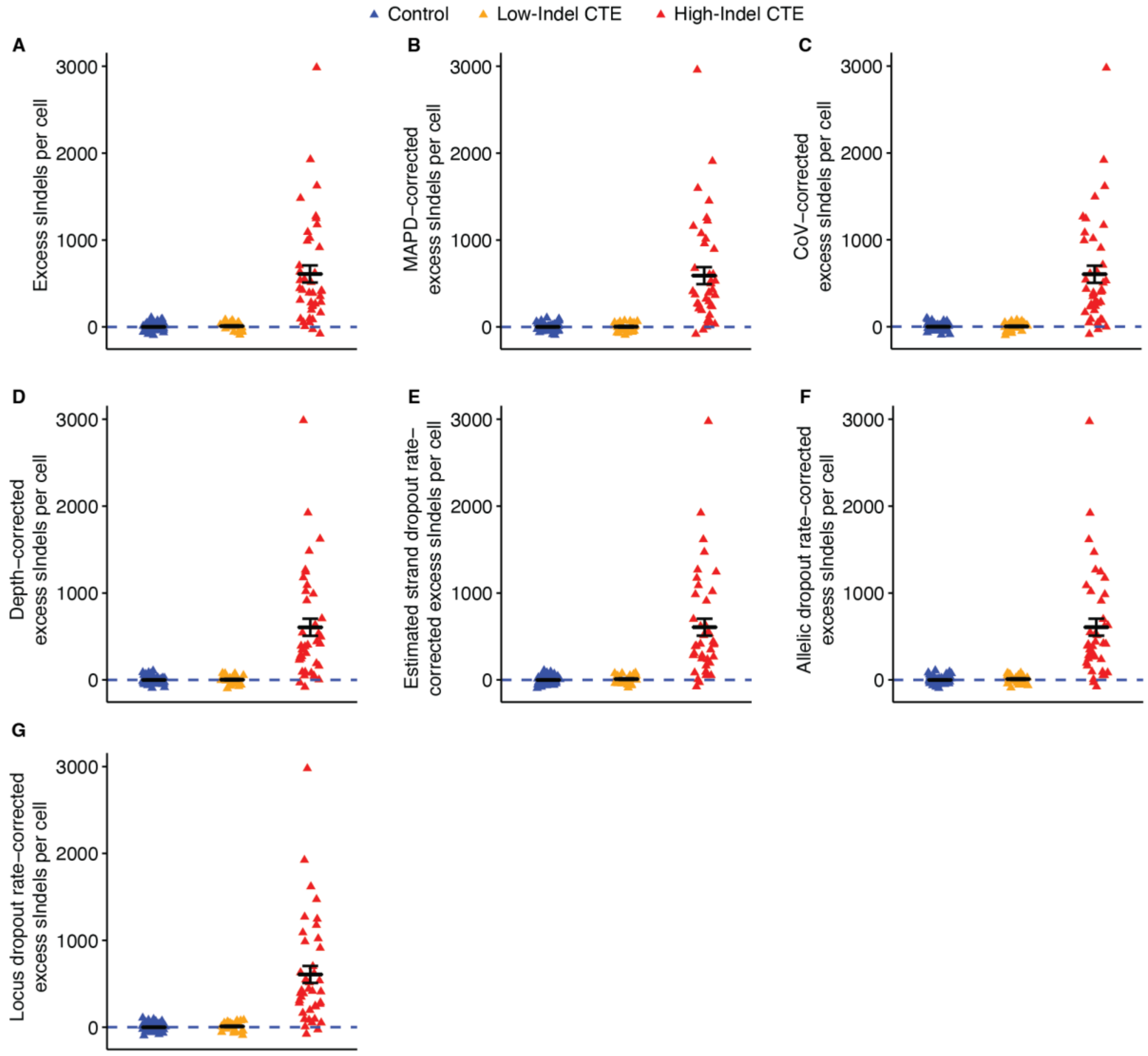

Fig. S10. Excess sIndels in neurotypical control (dark blue), Low-Indel CTE (yellow), and High-Indel CTE (red) after controlling for QC metrics. To evaluate the potential confounding effect of sample and sequencing quality on unadjusted excess sIndels (**A**), we calculated the excess sIndel burden corrected for five metrics (**B–G**). After controlling for MAPD (B), CoV (C), sequencing depth (D), estimated strand dropout rate (estimated as the square root of allelic dropout rate) (E), allelic dropout rate (F), and locus dropout rate (G), excess sIndels in High-Indel CTE and Low-Indel CTE are compared to neurotypical controls. Data are mean ± standard error. The dashed blue line shows sIndels attributable to age (zero excess).

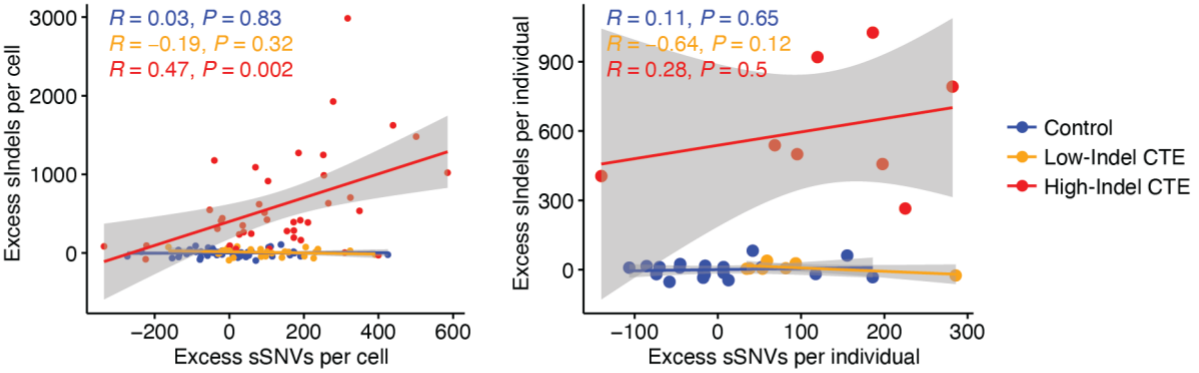

Fig. S11. Association between excess sIndels and excess sSNVs in CTE. Age-adjusted excess sIndels and sSNVs are correlated for each cell (*left*) and for each individual (*right*) in High-Indel CTE (red), Low-Indel CTE (yellow), and control (dark blue). Excess per individual was averaged across cells within the same individual. Pearson's correlation coefficient (*R*) and two-tailed p-value (*P*) are shown.

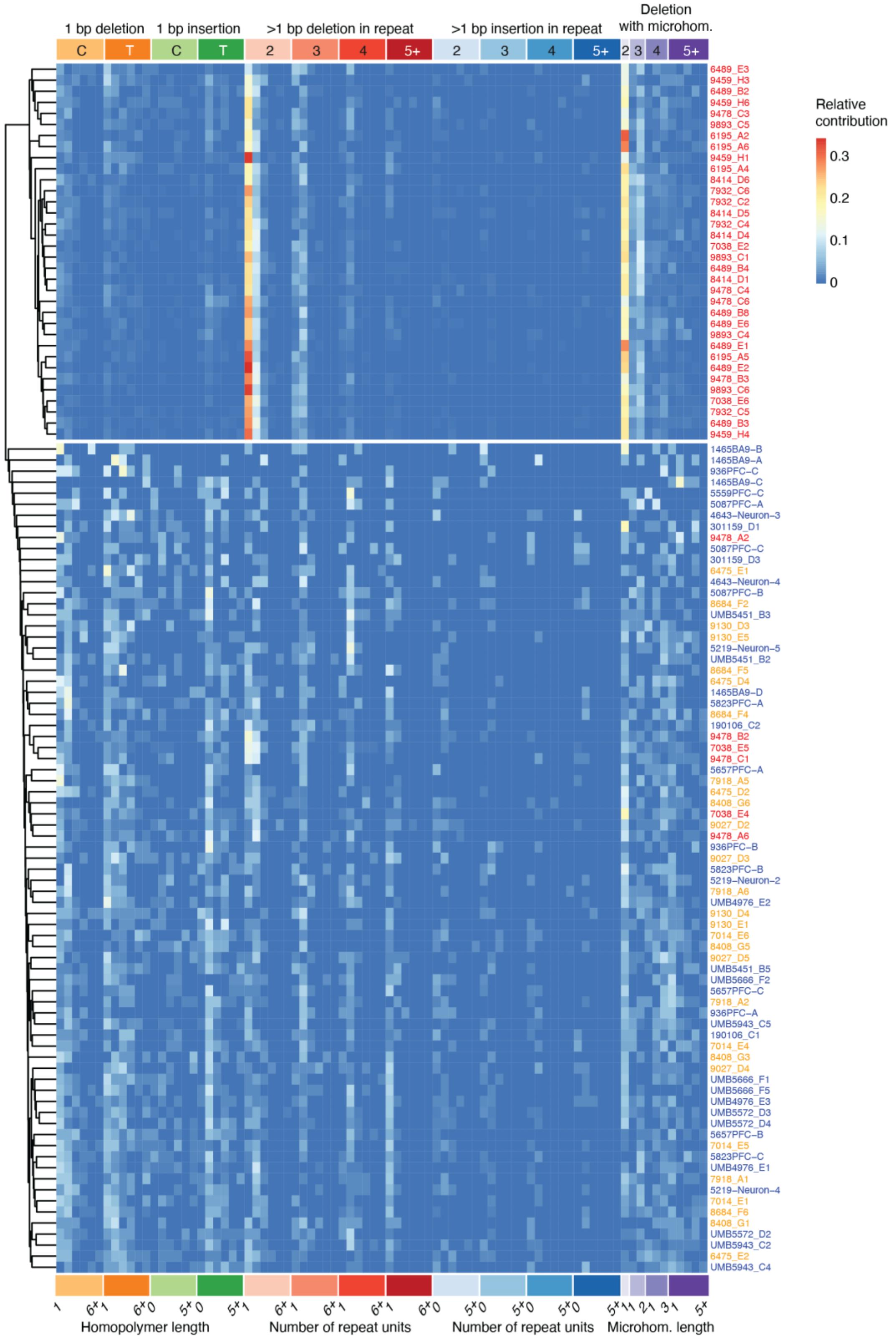

Fig. S12. Unsupervised clustering of PTA sIndel spectra for each neuron. PTA call sets from single neurons are separated into two clusters using UPGMA hierarchical clustering. Heatmap shows the proportion of 83 Indel contexts forming the ID83 spectrum on the columns and cells

on the rows, where cells from the same individual are colored based on the presence of excess sIndels in PTA data (Fig. 4A; High-Indel CTE: red, Low-Indel CTE: yellow, neurotypical control: dark blue). Cells from the top cluster represent High-Indel CTE, and cells from the bottom cluster predominantly represent Low-Indel CTE and neurotypical controls.

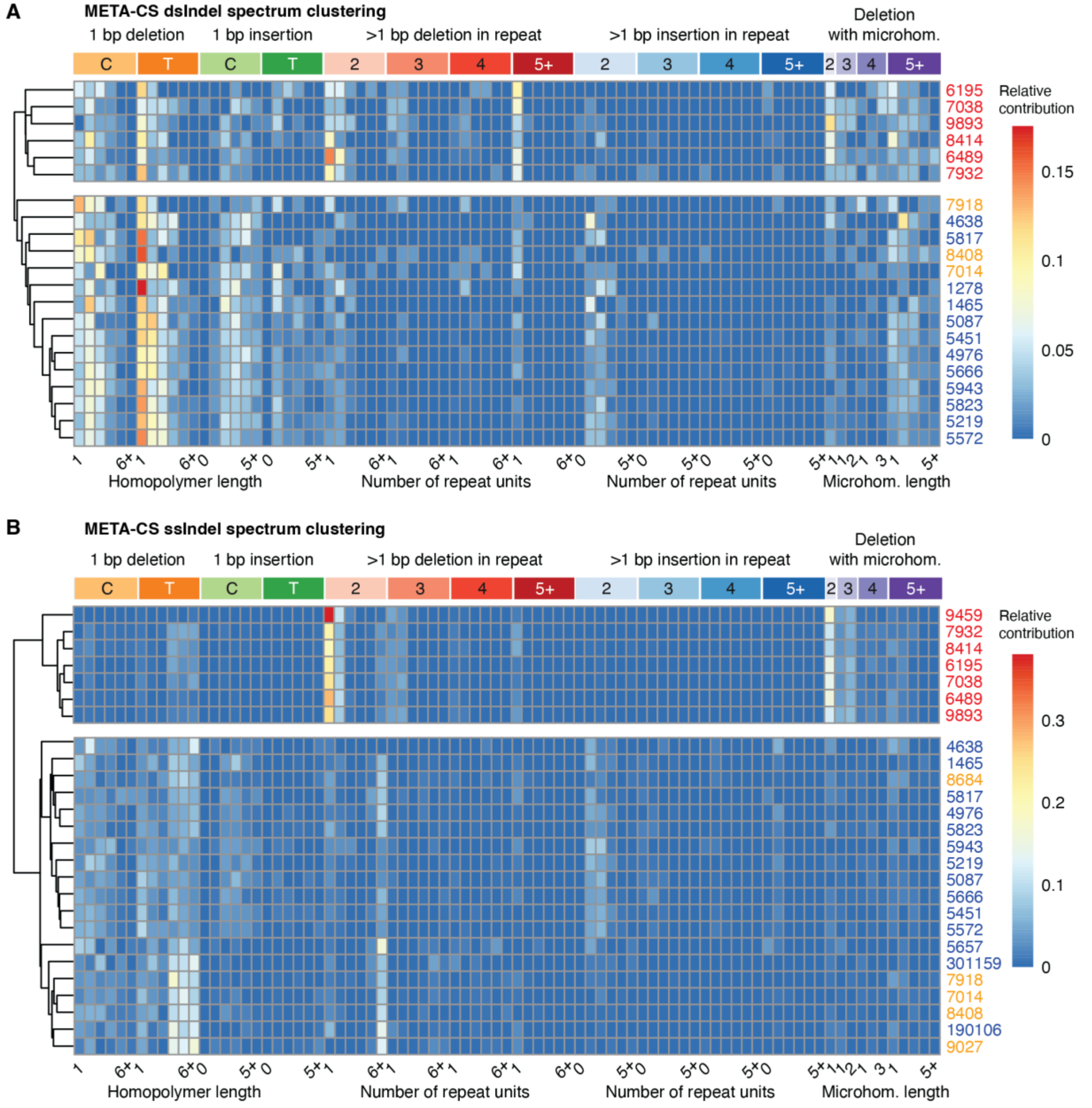

Fig. S13. Unsupervised clustering of META-CS dsIndel and ssIndel spectra for each individual. META-CS dsIndel (**A**) and ssIndel (**B**) call sets from single neurons are aggregated to generate individual-specific spectra before being separated into two clusters using UPGMA hierarchical clustering. Individuals with < 50 Indels are not shown. Heatmap shows the proportion of 83 Indel contexts forming the ID83 spectrum on the columns and individuals on the rows, where individuals are colored based on the presence of excess sIndels in their PTA data (Fig. 4A; High-Indel CTE: red, Low-Indel CTE: yellow, neurotypical control: dark blue).

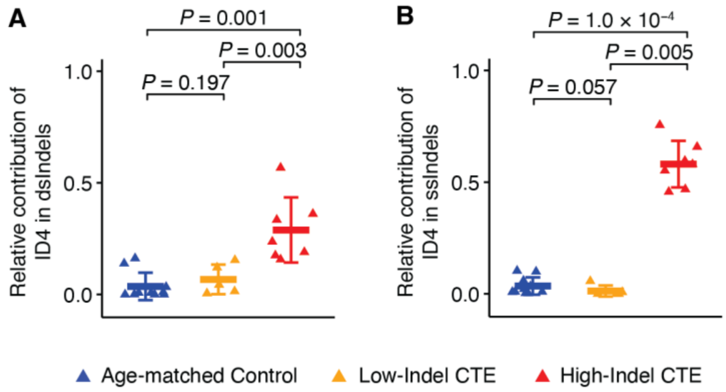

Fig. S14. Comparison of relative ID4 contribution to dsIndels and ssIndels across age-matched controls, Low-Indel CTE, and High-Indel CTE. Each triangle represents an individual from META-CS. ID4 contributions in dsIndels and ssIndels are compared across High-Indel CTE (red), Low-Indel CTE (yellow), and age-matched control (dark blue). Data are mean ± standard deviation. P-values are from two-tailed Wilcoxon tests.

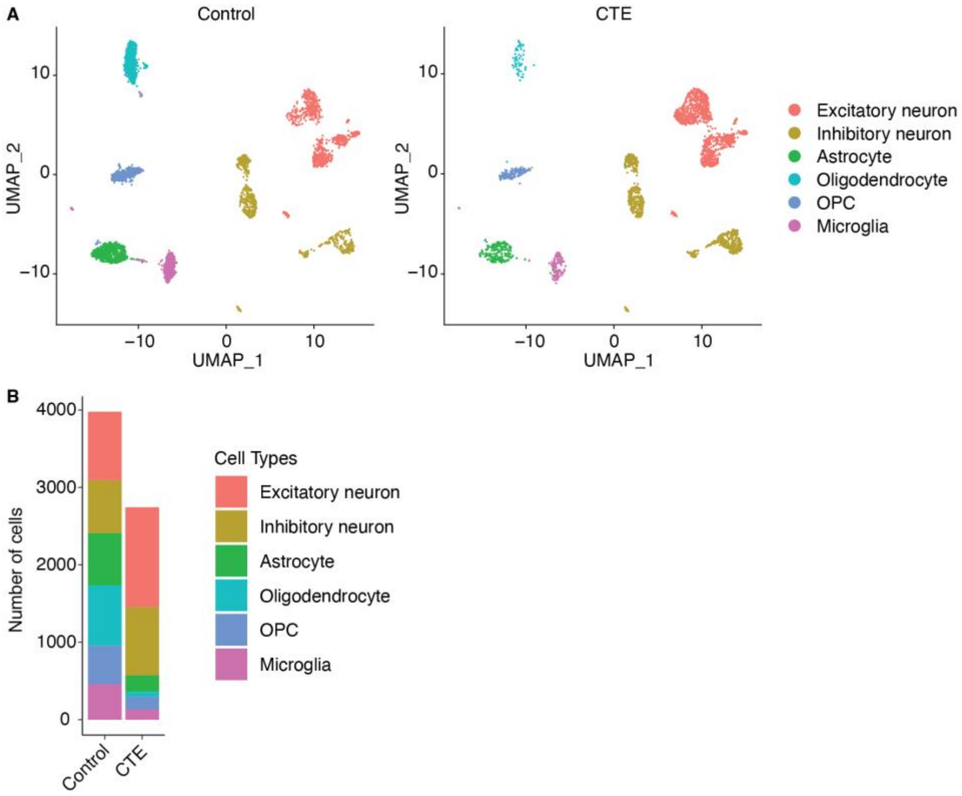

Fig. S15. Single-nucleus transcriptomic profiles of different cell types in neurotypical control and CTE samples. (**A**) Profiled cells from neurotypical control (*left*) and CTE (*right*) are plotted based on uniform manifold approximation and projection (UMAP) dimension reduction and colored by annotated cell types. (**B**) Number of cells of each cell type in neurotypical control and CTE, with a substantial number of neurons in both samples which are used for enrichment analyses.

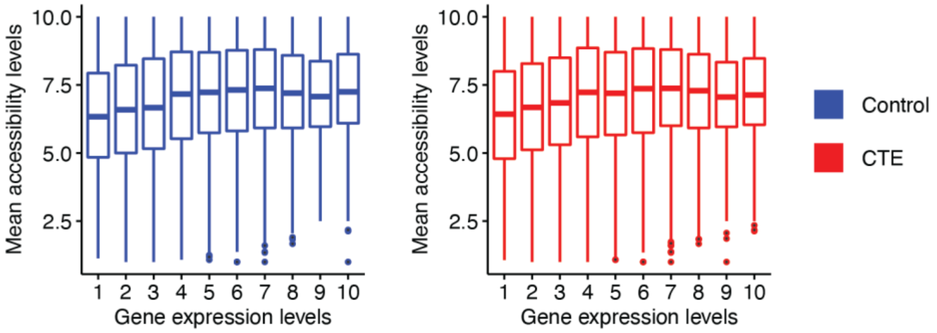

Fig. S16. Correlation of gene regions ranked based on gene expression levels and mean accessibility levels. For gene regions assigned to deciles 1 through 10 based on increasing gene expression levels in neurotypical control (*left*) and CTE (*right*), their mean accessibility levels are shown in box plots (bars from top to bottom show the first, second (median), and third quartile; whiskers extend 1.5 IQR with outlier data points shown separately).

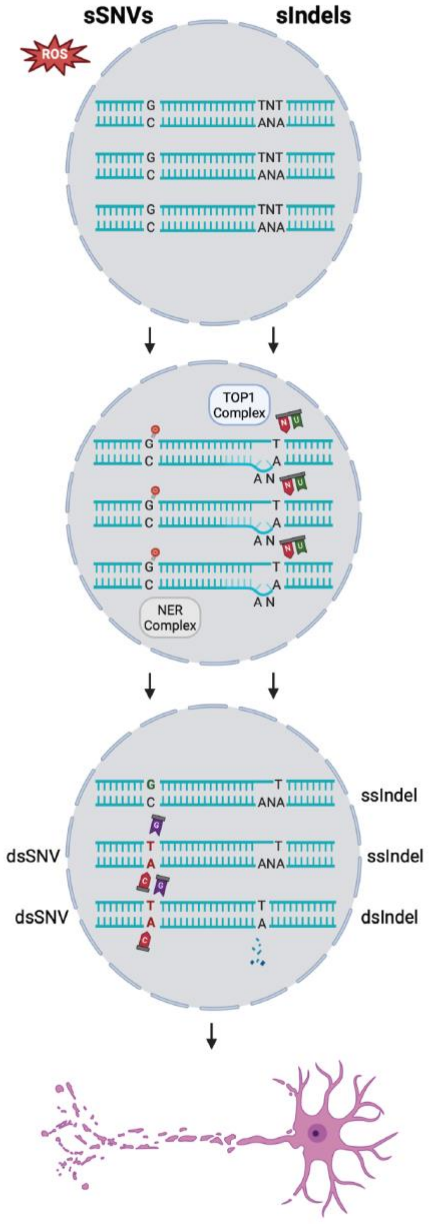

Fig. S17. Proposed mechanisms of mutagenesis for sSNVs and sIndels in CTE. An elevated DNA damage in CTE neuron in part caused by ROS leads to the accumulation of both sSNVs and sIndels. For sSNVs, oxidative damage frequently results in 8-oxoguanine on one strand which becomes a double-stranded C>A mutation when NER and other pathways fail to repair it. For sIndels, genome-embedded ribonucleotides are removed by TOP1-mediated activities during which a cleavage on one strand with the TNT motif leads to strand realignment and consequently a 2 bp single-stranded deletion. A portion of the single-stranded deletions may become double-stranded deletions potentially through other endogenous processes. The accumulation of such DNA damage may contribute to neurodegeneration and cell death.

Table S1. Sample information with number of neurons analyzed using two scWGS methods.

Case ID	Sex	Age (years)	Diagnosis	PTA Neurons	META-CS Neurons
**Neurotypical controls**					
**1278**	M	0.4	Normal	3	3
**5817**	M	0.6	Normal	3	16
**5871**	M	2	Normal	3	–
**4638**	F	15.1	Normal	3	4
**1465**	M	17.5	Normal	4	7
**5559**	F	19.8	Normal	3	–
**4643**	F	42.2	Normal	3	–
**5087**	M	44.9	Normal	3	5
**936**	F	49.2	Normal	3	10
**301159**	M	51	Normal	2	8
**5451**	M	57	Normal	3	9
**5666**	M	65	Normal	3	16
**190106**	M	67	Normal	2	10
**5943**	M	69	Normal	3	7
**5572**	F	70	Normal	3	6
**5219**	F	77	Normal	3	7
**5657**	M	82.2	Normal	3	8
**5823**	F	82.7	Normal	3	17
**4976**	F	104	Normal	3	13
				**56**	**146**
**CTE**					
**9130**	M	40	CTE (Stage III)	4	–
**7014**	M	47	CTE (Stage III)	4	10
**6195**	M	48	CTE (Stage III)	4	9
**8408**	M	49	CTE (Stage III)	4	10
**7932**	M	60	CTE (Stage III)	4	10
**7918**	M	68	CTE (Stage III)	4	10
**8684**	M	70	CTE (Stage III)	4	10
**9027**	M	71	CTE (Stage IV)	4	10
**6475**	M	73	CTE (Stage III)	4	–
**7038**	M	73	CTE (Stage IV)	4	10
**9459**	M	73	CTE (Stage IV)	4	8
**9478**	M	74	CTE (Stage IV)	8	–

**6489**	M	75	CTE (Stage IV)	8	10
**9893**	M	79	CTE (Stage III)	4	8
**8414**	M	82	CTE (Stage III)	4	10
				**68**	**115**
**RHI**					
**9316**	M	35	RHI	4	–
**7702**	M	62	RHI	4	–
**8592**	M	62	RHI	4	–
**6974**	M	73	RHI	4	–
				**16**	**–**
**AD**					
**1353**	F	57	AD (Braak VI)	4	–
**1647**	F	59	AD (Braak VI)	5	–
**2208**	F	69	AD (Braak VI)	4	–
**1456**	M	81	AD (Braak VI)	4	–
**2207**	M	83	AD (Braak VI)	3	–
**1995**	F	89	AD (Braak V)	3	–
**1828**	F	91	AD (Braak VI)	4	–
				**27**	**–**

Table S2.
Sample information. Clinical data, library and sequencing metrics.

Table S3.
PTA and META-CS sequencing statistics.

Table S4.
PTA SNV and Indel rates.

Table S5.
PTA SNV and Indel calls.

Table S6.
META-CS SNV and Indel calls.

Table S7.
Gene Ontology terms enriched for SNVs and Indels.
